# Prognostic analysis and validation of diagnostic marker genes in patients with osteoporosis

**DOI:** 10.3389/fimmu.2022.987937

**Published:** 2022-10-13

**Authors:** Xing Wang, Zhiwei Pei, Ting Hao, Jirigala Ariben, Siqin Li, Wanxiong He, Xiangyu Kong, Jiale Chang, Zhenqun Zhao, Baoxin Zhang

**Affiliations:** ^1^ Bayannur Hospital, Bayannur City, China; ^2^ Inner Mongolia Medical University, Hohhot, China; ^3^ The Second Affiliated Hospital of Inner Mongolia Medical University, Hohhot, China

**Keywords:** osteoporosis, geo, wgcna, immune cells, bioinformatics analysis

## Abstract

**Backgrounds:**

As a systemic skeletal dysfunction, osteoporosis (OP) is characterized by low bone mass and bone microarchitectural damage. The global incidences of OP are high.

**Methods:**

Data were retrieved from databases like Gene Expression Omnibus (GEO), GeneCards, Search Tool for the Retrieval of Interacting Genes/Proteins (STRING), Gene Expression Profiling Interactive Analysis (GEPIA2), and other databases. R software (version 4.1.1) was used to identify differentially expressed genes (DEGs) and perform functional analysis. The Least Absolute Shrinkage and Selection Operator (LASSO) logistic regression and random forest algorithm were combined and used for screening diagnostic markers for OP. The diagnostic value was assessed by the receiver operating characteristic (ROC) curve. Molecular signature subtypes were identified using a consensus clustering approach, and prognostic analysis was performed. The level of immune cell infiltration was assessed by the Cell-type Identification by Estimating Relative Subsets of RNA Transcripts (CIBERSORT) algorithm. The hub gene was identified using the CytoHubba algorithm. Real-time fluorescence quantitative PCR (RT-qPCR) was performed on the plasma of osteoporosis patients and control samples. The interaction network was constructed between the hub genes and miRNAs, transcription factors, RNA binding proteins, and drugs.

**Results:**

A total of 40 DEGs, eight OP-related differential genes, six OP diagnostic marker genes, four OP key diagnostic marker genes, and ten hub genes (TNF, RARRES2, FLNA, STXBP2, EGR2, MAP4K2, NFKBIA, JUNB, SPI1, CTSD) were identified. RT-qPCR results revealed a total of eight genes had significant differential expression between osteoporosis patients and control samples. Enrichment analysis showed these genes were mainly related to MAPK signaling pathways, TNF signaling pathway, apoptosis, and Salmonella infection. RT-qPCR also revealed that the MAPK signaling pathway (p38, TRAF6) and NF-kappa B signaling pathway (c-FLIP, MIP1β) were significantly different between osteoporosis patients and control samples. The analysis of immune cell infiltration revealed that monocytes, activated CD4 memory T cells, and memory and naïve B cells may be related to the occurrence and development of OP.

**Conclusions:**

We identified six novel OP diagnostic marker genes and ten OP-hub genes. These genes can be used to improve the prognostic of OP and to identify potential relationships between the immune microenvironment and OP. Our research will provide insights into the potential therapeutic targets and pathogenesis of osteoporosis.

## Introduction

Osteoporosis (OP) is a systemic bone disease characterized by low bone mass and destruction of bone microarchitecture (1), which increases the fragility of the bone and fracture risk (2, 3). OP is the fourth leading chronic disease after heart disease, dementia, and lung cancer (4). Bone homeostasis depends on osteoclast resorption and osteoblast formation, and an imbalance in this tightly coupled process can lead to the development of osteoporosis (5). Hip and vertebral fractures are two common osteoporotic fractures (6). Elderly patients with osteoporotic fractures often require hospitalization, resulting in poor quality of life, long-term medical care, disability, and even death (7). This creates a substantial economic and social burden worldwide and is a global public health challenge (8, 9). Due to its asymptomatic nature, patients with OP are often not diagnosed until the first osteoporotic fracture occurs. Therefore, it is very important to find biomarkers that enable early diagnosis.

In recent years, it has been established that bone and immune cells share the same progenitor cells and are affected by the same cytokines (3). They are functionally linked, and the infiltration of immune cells plays a vital role in the occurrence and development of OP (10). Factors such as the balance between Th1/Th2/Treg cells (3), inflammatory T cells (Th17) (4, 10), regulatory B cells (Bregs) (4), and macrophages (11) play an important role in regulating osteoblasts and osteoclast homeostasis, which in turn affects osteoporosis (12). Regarding the immune system, assessing the varying degrees of immune cell infiltration and identifying the compositional differences in the infiltrating immune cells can help elucidate the molecular pathological mechanism of OP and develop new immunotherapeutic targets.

In this study, to explore the potential diagnostic marker genes for OP, six OP diagnostic marker genes were screened for prognosis of OP, which can predict the prevalence of OP. First, the two OP immune signature subgroups were divided using a consensus clustering method, and differential expression analysis was performed to obtain 40 differentially expressed genes (DEGs). Secondly, eight OP-related differential genes were screened using Weighted gene co-expression network analysis (WGCNA), Gene Ontology (GO) functional annotation, Kyoto Encyclopedia of Genes and Genomes (KEGG) pathway enrichment, and Gene Set Enrichment (GSEA) analysis disease ontology (DO) disease annotation, and Gene Set Variation Analysis (GSVA) were performed on eight DEG. To conduct network analysis, we identified 10 hub genes using the cytoHubba function of Cytoscape software. RT-qPCR was performed on the plasma of osteoporosis patients and control samples. The results revealed a total of eight genes that had significantly different expression levels, and the following signaling pathways such asMAPK signaling pathway (p38,TRAF6) and NF-kappa B signaling pathway (c-FLIP, MIP1β), had significant different expressions. Out of the eight OP-related DEGs, six diagnostic marker genes were tested using Least Absolute Shrinkage and Selection Operator (lasso) regression and random forest using the new dataset. The Cell-type Identification by Estimating Relative Subsets of RNA Transcripts (CIBERSORT) algorithm was used to evaluate the level of immune cell infiltration in the two clusters, and the results showed that there were significant differences in proportions of monocytes, CD4 memory activated T cells, memory, and naïve B cells. Based on six OP-related diagnostic marker genes, two distinct molecular subtypes were identified using a consensus clustering approach. Prognostic analysis was carried out, and four key diagnostic marker genes were identified. Finally, the interaction network with miRNA, transcription factors (TF), RNA binding protein (RBP), and the drugs were constructed for key genes. Our study suggests that targeting these six diagnostic marker genes and ten hub genes may enhance the diagnosis and treatment of OP.

## Materials and methods

### Data downloaded

Download GSE56815 (13), GSE7158 (14) and GSE56116 (15) datasets from GEO database, In which GSE56815 contains 40 osteoporosis samples (OP) and 40 control samples from GPL96 sequencing platform, GSE7158 contains 12 osteoporosis samples Osteoporosis samples (OP) and 14 control samples were obtained from the GPL570 sequencing platform, and GSE56116 included 10 osteoporosis samples (OP) and 3 control samples from the GPL1433 sequencing platform, all of which were human peripheral blood sample. The above data were integrated for downstream analysis, the R package sva (16) was used to correct for batch effects between different datasets and log2 normalization was performed, and then the batch-corrected expression distribution was visualized using boxplots, where 62 osteoporosis samples and 57 control samples were included.

In order to analyze the expression of osteoporosis-related genes in all samples, we first obtained osteoporosis-related genes from the GeneCards database (17) through the keyword “Osteoporosis”, a total of 4657 genes, and intersected with the existing expression profiles. All 4657 genes were retained.

### Unsupervised clustering of samples

The R package factoextra (18) was used to determine the optimal number of clusters. The k-means clustering method was used for unsupervised clustering of all patients based on the optimal number of clusters, and the samples were divided into two categories. Finally, the R package was used to see the final clustering effect. Heatmap was used to visualize the gene expression profile of the two groups. The R package ggpubr (19) was used to construct the grouping histogram based on the sample clustering label. The Wilcoxon rank-sum test method was used to study the statistically significant differences between the groups. P<0.05 was considered to be statistically significant.

### Immune infiltration analysis

CIBERSORT is a deconvolution algorithm based on the principle of linear support vector regression to study the expression matrix of immune cell subtypes. It uses RNA-Seq data to estimate the abundance of immune cells in a sample (20). CIBERSORT: R package was used to estimate the quantity of 22 immune cells between disease and control samples in the datasets. The immune cell composition was visualized using boxplots. Differences in immune cell proportions were calculated using the Wilcoxon test, and P< 0.05 was considered statistically significant.

Pearson correlation was used to investigate the correlation between the immune cell expression in all patients. The two genes were correlated if the absolute value of the correlation coefficient > 0.3 and the P< 0.05. Correlations between matching gene pairs were plotted using the R package ggplot2 (21).

### Osteoporosis-related DEGs

In order to analyze the effect of different gene expression levels on patients with different subtypes of osteoporosis, the R package limma (22) was used to perform differential gene analysis between the two groups of patient samples in the integrated dataset. The significant differential genes (DEGs) were screened. Log2 (fold change) (log2FC) > 1.5 and Padj < 0.05 was set as the thresholds of DEGs. Genes with log2FC> 1.5 and Padj < 0.05 were up-regulated DEG, and genes with log 2FC <-1.5 and Padj < 0.05 were down-regulated DEG. The volcano plot shows the up-regulated DEG, and the R package pheatmap (23) shows the expression heat map of these DEG in all the samples. The R package ggpubr (19) was used to analyze the expression of osteoporosis-related genes in the two groups and construct grouped box plots based on the two subtype samples. Wilcoxon rank sum test method was used to test the statistically significant difference between the groups. P< 0.05 was considered statistically significant.

### Weighted gene co-expression network analysis

Weighted gene co-expression network analysis (WGCNA) is a systems biology method used to describe gene association patterns between different samples, and can be used to identify gene sets with highly coordinated changes. And identify candidate biomarker genes or therapeutic targets based on the interconnectivity of gene sets and the association between gene sets and phenotypes. We used the R package WGCNA (24) to calculate the key gene sets associated with the disease and normal two groups of samples and used them for subsequent analysis.

### Functional enrichment analysis

To investigate the biological differences between sample groups, gene set enrichment analysis (GSEA) was performed on DEG. Gene Ontology (GO) enrichment analysis is commonly used for large-scale functional enrichment analysis of genes at different dimensions and levels, mainly: biological process (BP), molecular function (MF), and cellular component (CC) (25). Kyoto Encyclopedia of Genes and Genomes (KEGG) pathway enrichment analysis is widely used for storing information about genomes, biological pathways, diseases, and drugs (26). Disease Ontology (DO) is the annotation of genes in the context of the disease. All the significantly DEG were subjected to GO, KEGG pathway enrichment, and disease annotation using the R package clusterProfiler (27) and the DOSE: R Package (28) to identify significantly enriched biological processes. The enrichment results were represented as bubble graphs for visualization. The significance threshold for the enrichment analysis was set at a corrected p-value < 0.05.

Gene Set Enrichment Analysis (GSEA) is a computational method used to determine whether a predefined gene set shows statistical differences between the two biological states. It is typically used to estimate expression in a dataset sample, changes in biological process, pathways, and activity (29). To investigate the differences in biological processes between the two groups of samples, based on the gene expression profiling dataset, the reference gene sets “c5.go.v7.5.1.entrez.gmt” and “c2.cp. kegg.v7.5.1.entrez.gmt” were downloaded from the Molecular Signatures Database MSigDB (30), for enrichment analysis and visualization of the dataset using the GSEA method included in the R package “clusterProfiler.” Adjusted p-values < 0.05 were considered statistically significant.

Gene Set Variation Analysis (GSVA) (31), is a non-parametric unsupervised analysis method. It mainly converts the gene expression matrix between different samples into the gene expression sets between samples. Quantity matrices were used to evaluate gene set enrichment results from microarray transcriptome data. To evaluate whether different pathways are enriched in different samples, the “c5.go.v7.5.1.entrez.gmt” and “c2.cp.kegg.v7.5.1.entrez.gmt” gene sets were retrieved from the MSigDB. Further, GSVA was performed at the gene expression level to calculate the differences in functional enrichment between groups (disease and control groups).

### Validation of osteoporosis marker genes

The identified diagnostic marker genes were validated using the osteoporosis dataset GSE7429 (32) retrieved from the GEO. The sequencing platform used for this dataset was GPL96 for humans. Data was first log-normalized and then divided into disease and control groups based on gene expression data of each marker. Lasso regression analysis was performed for univariate and multivariate analysis. The receiver operating characteristic (ROC) curve was used to evaluate the performance of marker genes in predicting the groups. ROC curves were drawn using the R package pROC (33).

### Network analysis

The Search Tool for the Retrieval of Interacting Genes/Proteins (STRING) (34) database searches for interactions between known and predicted proteins. The STRING database was used to select genes with a combined score greater than 400 to construct a protein-protein interaction (PPI) network related to DEG. Cytoscape (v3.7.2) (35) is used to visualize the PPI network model. PPI network analysis was performed using the CytoHubba (36) function in Cytoscape.

### Estimation of key genes

Ridge regression was first used to screen for osteoporosis-related genes. The analysis was performed using the R package glmnet (37) and was used to select the best lambda value. Only genes with coefficients other than zero were retained after regression analysis. The genes were further screened using logistic regression. The genes used to construct the model, and their corresponding coefficients were displayed in the form of forest plots using the R package forestplot (38).

To examine the multivariate influence of eigengenes in the diagnostic model, a new logistic multivariate regression model was constructed using the R package rms (39) on the genes with significant absolute weights in the previous model. To verify the predictive grouping efficacy of key genes, the ROC package pROC (33) was used to draw the ROC curve of the model and calculate the area under the curve (AUC).

### Panorama of key genes

The R package RCircos (40) was used to map the location of genes on the chromosomes. The chromosome data were provided by the R package, and the information regarding the location of genes on chromosomes was downloaded from the ENSEMBL (41) database. Boxplot was constructed using R package ggplot2 to analyze the differences in the expression of key genes in all the patients. The Gene Expression Profiling Interactive Analysis (GEPIA2, 42) explores the RNA-seq expression data from tumor and normal tissues retrieved from TCGA and GTEx databases. GEPIA2 was used to obtain the expression of key genes in tumor and normal samples from various human tissues.

### Multidimensional network analysis of key genes

TF controls gene expression by interacting with target genes at the transcriptional stage. miRNet database (43) was used to construct the regulatory network of key genes, TFs, and miRNAs. RBP is an important protein of the cells, which interacts with RNA by recognizing certain RNA binding domains. It is widely involved in RNA splicing, transport, sequence editing, intracellular localization, translation control, and post-transcriptional regulation. The regulatory network of key genes-RBP was constructed using the RBP2GO database (44). RNAactDrug database (45) was used to build a key gene-drug regulatory network.

### Real-time fluorescence quantitative PCR

Peripheral blood of four clinical osteoporosis patients and three healthy adults were obtained from the Second Affiliated Hospital of Inner Mongolia Medical University. (This study was performed in line with the principles of the Declaration of Helsinki. Approval was granted by the Ethics Committee of Second Affiliated Hospital of Inner Mongolia Medical University. The ethical review number: YKD202002055). 5ml peripheral venous blood was collected with EDTA-K2 anticoagulant blood collection tube. After centrifugation at 1500 r/min for 15 minutes, the uppermost plasma was obtained. Total RNA was extracted from plasma samples. The genomic DNA was removed from the RNA sample, and RNA was reverse transcribed using the PrimeScript™ RT reagent Kit with gDNA Eraser (RR047A, Takara, Japan). Real-Time quantitative PCR was performed using the SYBR^®^ Premix Ex Taq (Takara, Japan, RR820A) kit using a real-time PCR machine (ABI-7500, Applied Biosystems, USA). The PCR amplification was carried out for a total of 42 cycles. The mean + standard error of three independent experiments were calculated, with each experiment repeated three times. Relative mRNA expression levels were calculated using *GAPDH* as an internal reference.

### Statistical analysis

All data processing and analysis were conducted using R software (version 4.1.1). The student’s t-test was used to compare the two continuous variables groups and evaluate the statistical significance of normally distributed variables. The independent and the differences among non-normally distributed variables were analyzed using the Mann-Whitney U test (i.e., the Wilcoxon rank sum test). Chi-squared test or Fisher’s exact test was used to compare and analyze statistical significance between two groups of categorical variables. Correlation coefficients between different genes were calculated using Pearson correlation analysis. The t-test was used to compare the mean values of two groups of samples, and the analysis of variance (ANOVA) test was used to compare the mean values of multiple groups of samples. All statistical P values were two-sided. P < 0.05 considered statistically significant.

## Results

### Gene Expression Omnibus data preprocessing

**Figure 1:**
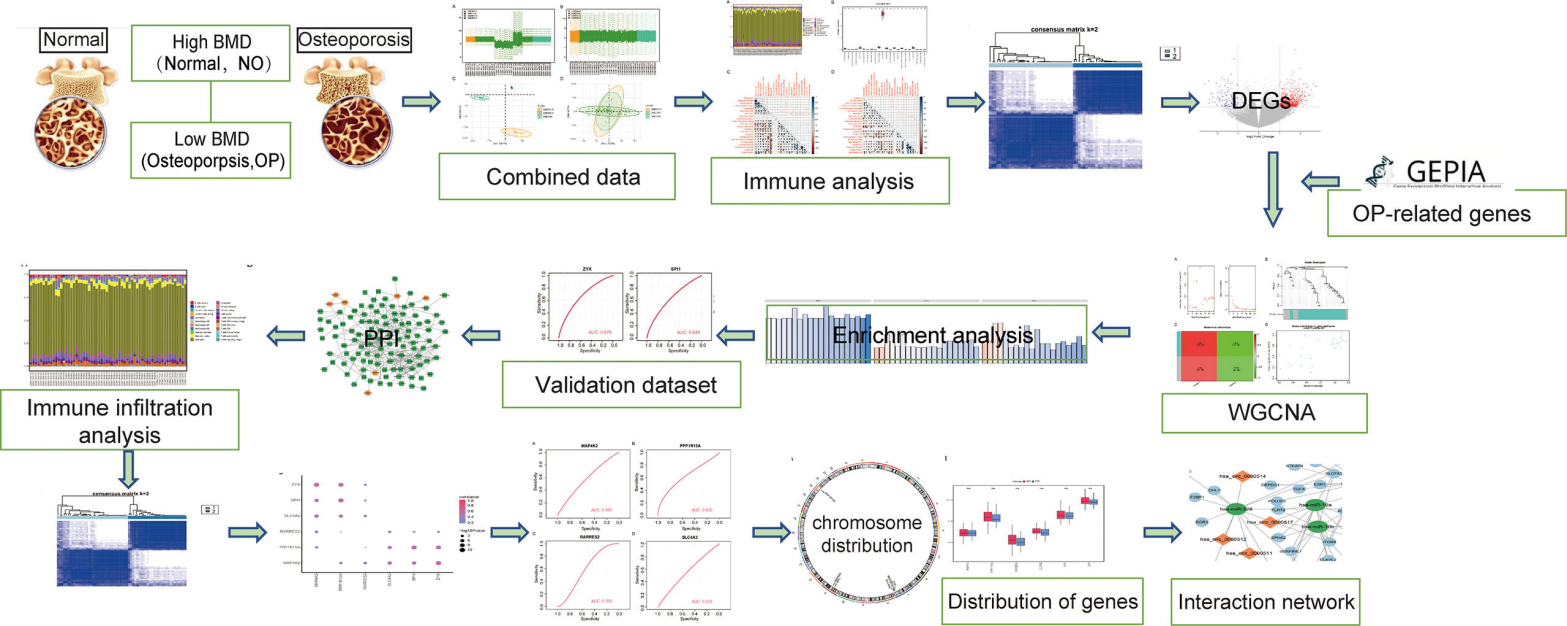
Flowchart.

**Figure 2:**
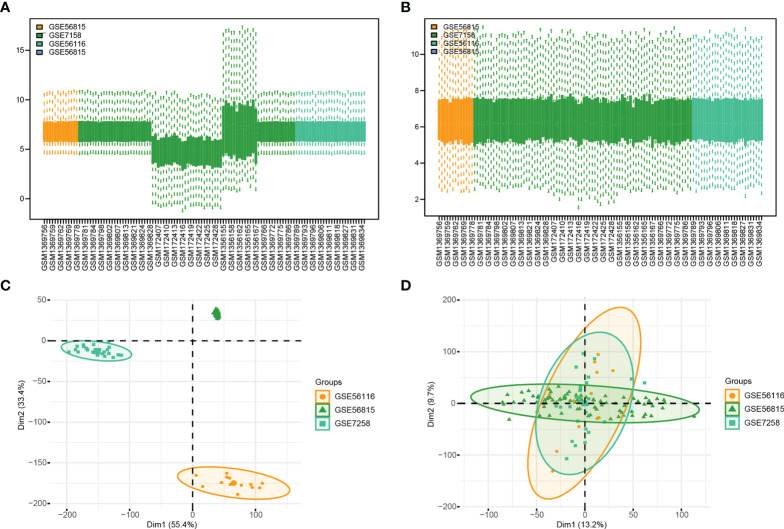
Gene Expression Omnibus (GEO) data preprocessing. **(A, B)** are the differences in data distribution before and after data set processing. **(C, D)** results from PCA dimensionality reduction before and after data set processing.

In order to clearly show the specific process of this study, the bioinformatics analysis process is specially summarized as shown in the figure (**Figure 1**). To construct a panorama of osteoporosis-related genes in all samples, the expression profiles of all three datasets were integrated. Datasets from different sources generally have severe batch effects. Hence the raw data was first analyzed and then corrected for batch effects and log normalization. Boxplots were drawn using the data OP and Normal groups retrieved from datasets GSE56815, GSE7158, and GSE56116 (**Figures 2A, B**). The results show that after batch correction and log normalization, the distribution of expression profiles of all the samples tends to be overall consistent., which was more conducive to improving the accuracy and robustness of the downstream analysis (**Figure 2**). The batch effect was removed to obtain an integrated dataset, which included 62 osteoporosis and 57 control samples.

### Overall immune level analysis and differential analysis of immune signature subtypes in osteoporosis

**Figure 3:**
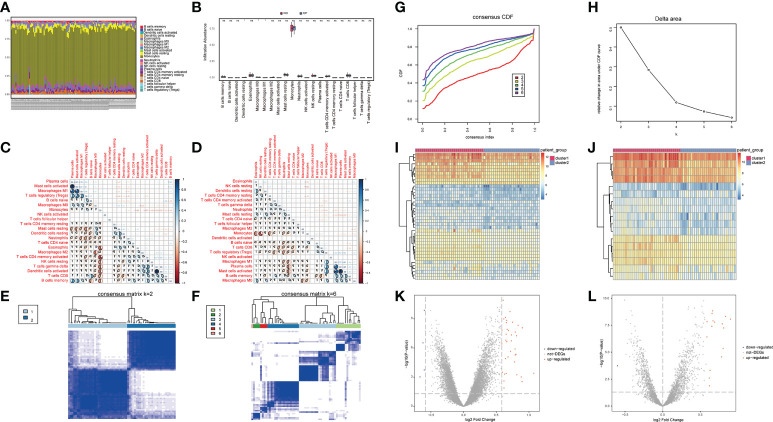
Overall immune level analysis and differential analysis of immune signature subtypes in osteoporosis. **(A)** The content of immune cells between the osteoporosis (OP) and the control group. Different colors represent different immune cells, and the horizontal axis represents the patient id. **(B)** Histogram of immune cell content, the horizontal axis represents immune cells, the vertical axis represents cell content, red represents the control group samples, and blue represents the disease group samples. 3C-D: Correlation of immune cell content in the normal group **(C)** and disease group samples **(D)**; red indicates a negative correlation, and blue indicates a positive correlation. **(E, F)** Consistent clustering result graph, different colors represented different groups. **(G)** Cumulative Distribution Function (CDF) plot of consensus clustering, showing the curve of the CDF as the number of clusters changes. **(H)** Delta Area plot, calculating the relative change in the area under the curve (AUC) of the CDF as the number of clusters increases. **(I, J)** Heatmaps of differentially expressed genes (DEG), where red is for cluster 1 and blue is for cluster 2. **(K, L)** the volcano plot for DEG, the abscissa is log2FoldChange, the ordinate is -log10 (adjust P-value), red nodes indicate up-regulated DEG, gray nodes indicate genes that are not significantly differentially expressed, and blue nodes indicate down-regulated genes DEG.

The immune microenvironment is a complex integrated system composed mainly of immune cells, inflammatory cells, fibroblasts, interstitial cells, various cytokines, and chemokines. The analysis of infiltrated immune cells in samples plays an important role in understanding the pathology, prognosis, and treatment of the disease. To analyze the differences in immune levels between normal and disease states, we analyzed the overall immune profile of normal (NO) and osteoporotic (OP) patient samples (**Figures 3A–D**). CIBESORT analysis of immune infiltration analysis (**Figure 3A**) reveals that the content of monocytes was high in OP and normal samples. Compared to normal samples, the OP patient samples only showed significant differences in the expression levels of M0, M1 Macrophages, and activated dendritic cells (**Figure 3B**). Further, the correlation of immune cell content in normal and OP patient samples was analyzed. The results revealed a significant correlation between the memory B cells and monocyte content in normal samples and various immune cells (**Figure 3C**). In OP patient samples, a significant correlation between the content of the activated mast cells and M0 macrophages, and various other immune cells (**Figure 3D**).

Here, a consensus clustering method commonly used in tumor typing is used. We wanted to use this method to divide the 62 OP patient samples from the 3 datasets into an appropriate number of subgroups. Furthermore, differential expression analysis was performed on its different subgroups. The obtained differential expression results can not only represent the difference between OP and normal samples, but also reflect the differential genes between different types (or grades) of OP. Different expression patterns were identified in 62 osteoporosis patient samples using a consensus clustering method (ConsensusClusterPlus package in the R software). **Figures 3E, F** show the matrix heatmaps for k=2 and k=6, and the clustering results are better separated when k=2. Secondly, considering the consistent Cumulative Distribution Function (CDF) plot and the Delta Area Plot, the CDF at k=2 had a lower slope of decline and a lower change in the AUC (**Figures 3G, H**). Two osteoporosis subtypes (cluster1 and cluster2) were finally identified (**Figures 3E–H**), with cluster 1 containing 34 samples and cluster 2 containing 28 samples. (**Figure 3E**).

To understand the biological differences between the two patient subgroups, the DEG analysis was first performed on the two patients’ subgroups. The threshold was set as padj<0.05 and foldchange>1.5 or foldchange<-1.5. and a total of 40 DEGs were identified during the analysis. There were 36 up-regulated genes and four down-regulated genes (**Figures 3I, K**). The OP-related genes retrieved from GeneCards were intersected, and a total of 17 genes that were significantly different between the two groups of patients and related to OP were retained (**Figures 3J, L**).

### Functional enrichment analysis between samples

To explore the relationship between the differentially expressed OP-related genes, WGCNA analysis was performed on the DEG between the two groups of patients (**Figure S1A**). A co-expression module was identified (**Figure S1B**), and the gene set with the highest correlation was identified and subjected to subsequent analysis (**Figures S1C, D**) 1198 key genes were obtained. After intersecting with differentially expressed OP-related genes, eight genes were obtained for subsequent analysis (**Table S1**).

**Figure 4:**
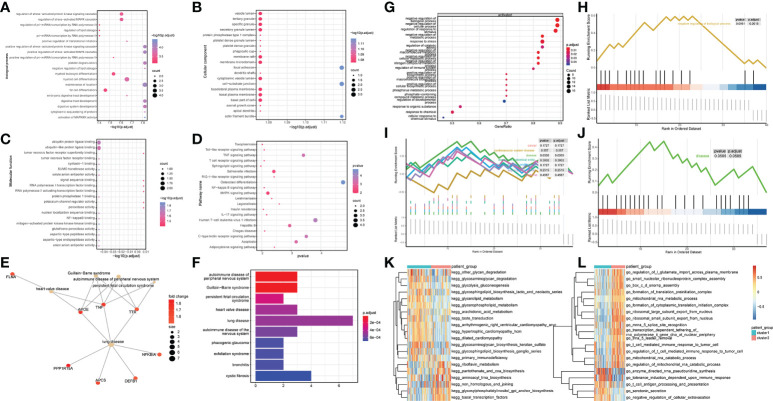
DEGs identification and functional enrichment analysis between samples. **(A-C)** GO enrichment analysis was performed on up-regulated, and down-regulated genes, and biological process (BP), molecular function (MF), and cellular component (CC) were displayed. Node size indicates the number of genes enriched in the pathway, and node color indicates -log10 (p-value). **(D)** Results of KEGG pathway analysis, the node size represents the number of genes enriched in the pathway, and the node color represents the p-value. **(E, F)** DOSE enrichment results. **(G)** Overall Gene Ontology enrichment analysis results. **(H)** gsea-go enrichment pathway analysis results. **(I, J)** The overall and partial gsea_dose enrichment results are displayed. **(K, L)** GSVA enrichment results.

To explore the influence of the differential genes on the biological functions of different patient subtypes, GO enrichment analysis was performed on the differential genes. The biological processes enriched by these differential genes were myeloid cell differentiation, regulation of stress-activated MAPK cascade (**Figure 4A**), and cell groups such as vesicle lumen and tertiary granule (**Figure 4B**). The molecular functions enriched by these differential genes were ubiquitin protein ligase binding and ubiquitin-like protein ligase binding (**Figure 4C**). KEGG pathway enrichment analysis revealed these differential genes significantly enriched pathways such as Salmonella infection and MAPK signaling pathway (**Figure 4D**). In addition, Disease ontology analysis (DOSE) was performed on the differential genes. The results revealed that the DEG between different patient subtypes was significantly enriched in heart valve disease (**Figures 4E, F**).

GSEA analysis was performed on the differential genes, and the results revealed significant differences in the following biological processes between the two groups of patient samples. In cluster 1 patient samples, the regulation of response to stimulus, negative regulation of the cellular process, and negative regulation of biological and other biological processes were activated (**Figures 4G, H**). Simultaneously, among the seven related pathways annotated by DOSE, the disease pathway was significantly annotated (**Figures 4I, J**).

The results of GSVA analysis showed that go_chloride_transprt was activated in the cluster 2 patient samples, and in the cluster 1 patient samples, kegg_ubiquitin_mediated_proteolysis was inhibited (**Figures 4K, L**).

### Validation of diagnostic marker genes in a new dataset

**Figure 5:**
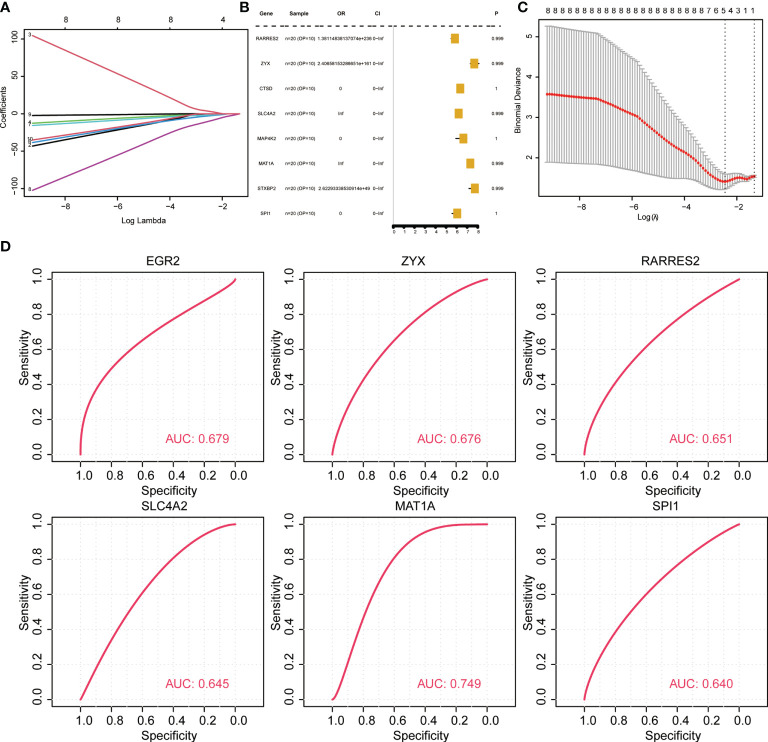
Validation of diagnostic marker genes in a new dataset. **(A, C)** lasso regression analysis results. **(B)** Univariate analysis results. **(D)** ROC curve, indicating the diagnostic performance of the genes.

To evaluate if the key identified genes could serve as diagnostic marker genes for osteoporosis and to test the robustness of the predicted diagnostic marker genes, new datasets (GSE7429, GPL96) were retrieved from GEO. The data were preprocessed consistently, and the association of genes with OP was first tested using lasso regression and random forest. Univariate analysis (**Figures 5A–C**) revealed that none of the eight key genes were significant associated (EGR2, RARRES2, ZYX, SLC4A2, MAP4K2, MAT1A, PPP1R15A, SPI1). Hence, the performance of key genes in predicting disease samples was evaluated by plotting the ROC curves of the key genes. There were eight key genes related to OP differences. ROC curves of each of the eight key genes associated with OP in normal and OP tissue samples were constructed. The results revealed six key genes with predicted AUC > 0.6, namely RARRES2, ZYX, SLC4A2, EGR2, MAT1A, and SPI1 (**Figure 5D**). The results show that these six genes could successfully distinguish between OP and normal samples.

### Verification of Hub genes and signaling pathway molecules in clinical samples

To understand the relationship between the DEG-related to OP in the biological network, eight OP-related differential genes were analyzed using WGCNA, and 11 PPI regulatory networks were downloaded from the STRING database (**Figure S2A**). The regulatory relationship was imported into Cytoscape for network analysis, and the top ten hub genes were identified (**Figure S2B**). Hub genes were TNF, RARRES2, FLNA, STXBP2, EGR2, MAP4K2, NFKBIA, JUNB, SPI1, CTSD. **Figure S2C** shows the diagnostic genes that distinguish OP samples from control samples scattered in the PPI network, further reiterating that the research focus was still on validating the diagnostic genes.

**Figure 6:**
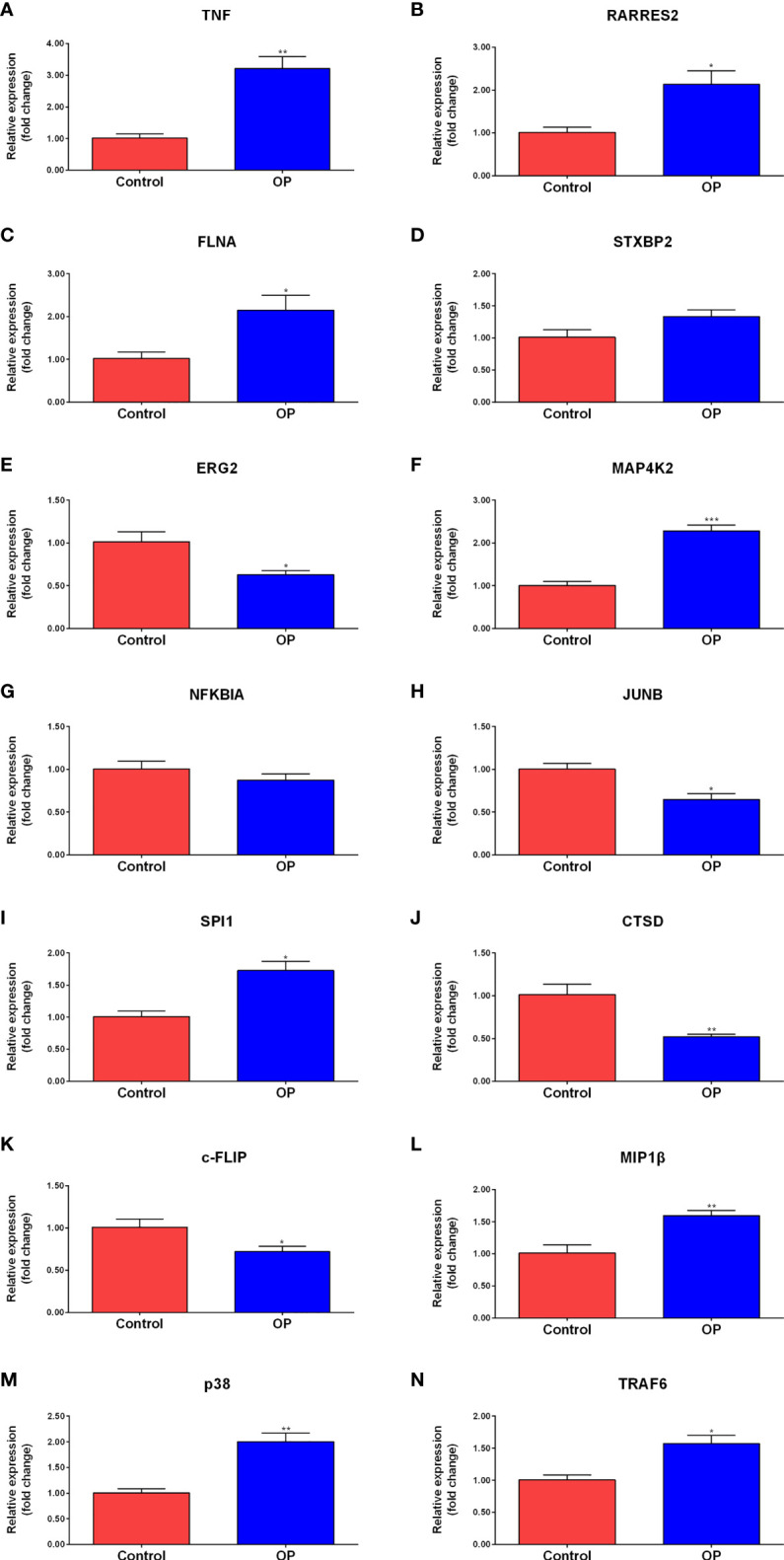
RT-qPCR results of the expression level of Hub genes and signaling pathway molecules. Comparison of mRNA expression levels of 10 Hub genes and 4 signaling pathway molecules in plasma of control group (n=3) and OP group (n=4). Among them, the mRNA expression levels of TNF **(A)**, RARRES2 **(B)**, FLNA **(C)**, MAP4K2 **(F)**, SPI1 **(I)**,MIP1b **(L)**, p38 **(M)**, and TRAF6 **(N)** in the plasma of the OP group were significantly increased. the mRNA expression levels of EGR2 **(E)**, JUNB **(H)**, CTSD **(J)** and c-FLIP **(K)** were significantly decreased in the OP group. There was no significant difference in the mRNA expression levels of STXBP2 **(D)** and NFKBIA **(G)** between the two groups. P-values were calculated using a two-sided unpaired Student’s t-test. (*P < 0.05, **P < 0.01,***P < 0.001 vs. Control).

The peripheral blood from clinical samples was collected to explore the expression of hub genes. The mRNA expression of TNF, RARRES2, FLNA, STXBP2, EGR2, MAP4K2, NFKBIA, JUNB, SPI1, and CTSD in the plasma of the control group and the OP group was detected (**Figures 6A–J**). The RT-qPCR results showed that compared to the control group, the mRNA expression levels of TNF, RARRES2, FLNA, MAP4K2, and SPI1 in the plasma of the OP group were significantly increased, the mRNA expression levels of EGR2, JUNB, and CTSD were significantly decreased in the OP group. There was no significant difference in the mRNA expression levels of STXBP2 and NFKBIA between the two groups.

To further explore the expression of MAPK and NF-kappa B signaling pathway-related genes, the peripheral blood from clinical samples was collected, and total RNA was extracted to study the expression levels of c-FLIP, MIP1β, p38, and TRAF6 in the plasma of the control group (Control) and the osteoporosis group (OP) (**Figures 6K–N**). The results of RT-qPCR showed that compared to the control group, a significant decrease in the mRNA expression level of c-FLIP in the plasma of the OP group was observed, and a significant increase in the mRNA expression levels of MIP1β, p38, and TRAF6 was observed.

### Immune infiltration analysis and molecular subtype construction based on key OP-related diagnostic marker genes

**Figure 7:**
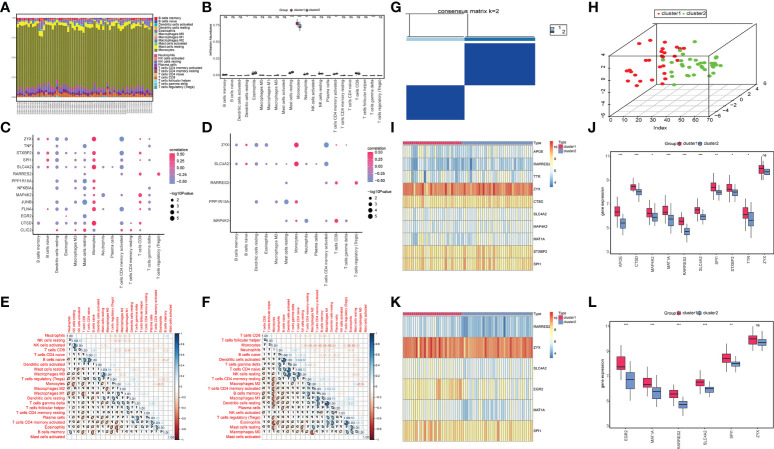
Immune infiltration analysis and molecular subtype construction based on key OP-related diagnostic marker genes. **(A)** The accumulation of immune cells between cluster 1 and cluster 2, different colors represent different immune cells, and the horizontal axis represents the patient id. **(B)** Histogram of immune cell content, the horizontal axis represents immune cells, the vertical axis represents cell content, red represents cluster 1 samples, and blue represents cluster 2 samples. **(C)** Correlation diagram between OP differentially expressed genes and immune cells, the horizontal axis represents immune cells, the vertical axis represents genes, the color of nodes represents the size of the correlation, and the size of the nodes represents the level of significance. **(D)** Correlation diagram between key genes and immune cells, the horizontal axis represents immune cells, the vertical axis represents key genes, the color of the nodes represents the size of the correlation, and the size of the nodes represents the level of significance. **(E, F)** Correlation of immune cell content in cluster 1 **(E)** and cluster 2 samples **(F)**; red indicates a negative correlation, and blue shows a positive correlation. **(G)** Graph of Consistent clustering results. **(H)** PCA analysis of cluster1 and cluster2. **(I, J)** Heat map **(I)** and box plot **(J)** shows the expression of OP-related differentially expressed genes between the two groups. **(K, L)** Heatmap **(K)** and boxplot **(L)** show the expression levels of key genes between the two groups. Red represents cluster1, and blue represents cluster2. (*P < 0.05, **P < 0.01, ***P < 0.001, ns P > 0.05 no significance vs. Control).

The CIBERSORT results showed (**Figure 7A**) that the monocyte content was significantly high in the two groups of OP patients. Compared to cluster 1, OP patients in cluster 2 had lower levels of CD8 in Monocytes and T cells (**Figure 7B**). OP patients in cluster 1 had a low content of activated dendritic cells, resting mast cells, neutrophils, and activated CD4 memory T cells compared to cluster 2 (**Figure 7B**). The correlation between differentially expressed characteristic genes related to OP, the diagnostic marker genes, and immune cell content was analyzed. The results showed a significant positive correlation between the expression levels of monocytes and various differentially expressed characteristic genes related to OP (**Figure 7C**). Monocytes positively correlated with multiple diagnostic marker genes, and a negative correlation was observed between activated CD4 memory T cells and multiple diagnostic marker genes (**Figure 7D**). Simultaneously, the correlation between the immune cell content of samples from patients in cluster 1 and samples from patients in cluster 2 was calculated. The results showed a significant correlation between the content of the memory B cells and various immune cells in samples from cluster 1 (**Figure 7E**). The content of monocytes in the samples significantly correlated with the content of various other immune cells (**Figure 7F**).

Based on the six OP-related diagnostic key genes, two different molecular subtypes and two patient subgroups (cluster1 and cluster2) were identified (**Figure 7G**) using a consensus clustering method (“ConsensusClusterPlus” package in the R software). Cluster 1 contained 28 samples, and cluster 2 had 34 samples. The PCA clustering results showed significant differences between the two clusters (**Figure 7H**).

The heatmaps and box plots were constructed based on the expression to observe changes in isoforms and gene expression. WGCNA analysis revealed a significant increase in the OP-related DEG in cluster1 (**Figures 7I, J**). Simultaneously, the expression of diagnostic marker genes in cluster1 was significantly higher compared to cluster2 (**Figures 7K, L**).

### Key gene correlation analysis based on osteoporosis subtypes

**Figure 8:**
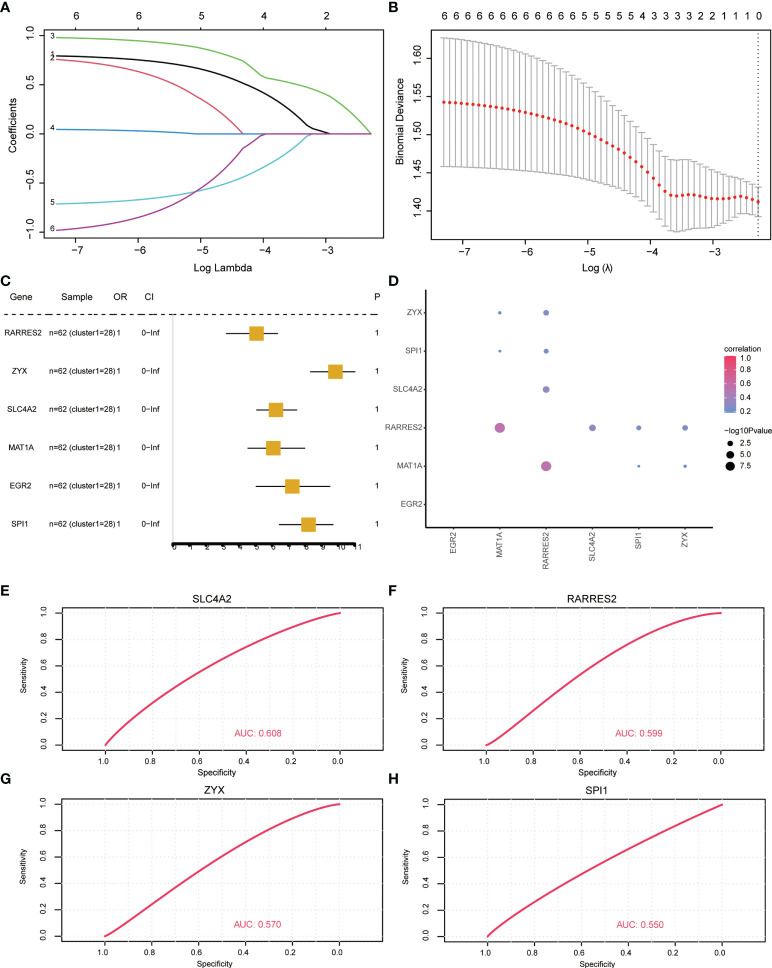
Key gene correlation analysis based on osteoporosis subtypes. **(A, B)** Lasso regression analysis results. **(C)** Univariate analysis results. **(D)** Similarity between key genes, the size of the point represents significance; the larger the point, the more significant the color indicates the correlation, and the redder the color, the more relevant. **(E–H)**: ROC curve, indicating the diagnostic performance of the genes.

To analyze the influence of key diagnostic genes on patients with different subtypes of OP, logistic univariate regression analysis was used to identify six genes with poor influence on OP (**Figure 8C**). Coefficients for six genes were calculated based on LASSO analysis (**Figures 8A, B**). The correlation between the expression of key genes was calculated, and the RARRES2 gene showed a significant positive correlation with multiple other genes (**Figure 8D**).

To analyze whether the key genes could better distinguish the two molecular subtypes, the gene expression was multiplied by the corresponding coefficient and added to establish the OP prediction score. The final prediction score of each sample was calculated. The results revealed that these four genes (RARRES2, SLC4A2, SPI1, ZYX) could better predict different subtypes of OP patients (**Figures 8E–H**).

## Discussion

In recent years, due to the lack of reliable early diagnostic tools and methods, most OP patients have suffered pathological fractures, which would require internal fixation and surgical interventions. This causes causing severe physical, mental, and economic burdens to the patients. Previous studies have shown that the immune microenvironment may play an important role in the occurrence and development of OP (4, 11, 46). However, the specific targets and therapeutic mechanisms of OP remain unclear and require further investigation. Our study screened 40 DEGs, eight OP-related differential genes, ten hub genes, six OP diagnostic marker genes, and four OP diagnostic marker key genes. Furthermore, the correlation between OP prognostic models and immune signatures and immune cell infiltration profile revealed that the immune microenvironment might be involved in the pathogenesis of OP.

WGCNA analysis screened eight OP-related differential genes. GO, KEGG, GSEA, DOSE, and GSVA enrichment analysis was also performed. The results showed that OP-related genes mainly enriched the MAPK signaling pathway, TNF signaling pathway, apoptosis, and Salmonella infection. RT-qPCR results showed significant differences in the MAPK signaling pathway (p38, TRAF6) and NF-kappaB signaling pathway (c-FLIP, MIP1β). Previous studies have shown that OPG/RANK/RANKL (9), IL-1β (47),TRAF6 (48), NFATc1, OSCAR and NF-κB (49), and other genes related to apoptosis, inflammation, and osteogenic differentiation (6). These genes regulate bone metabolism *via* the MAPK signaling pathway and TNF signaling pathway, which affects OP. Salmonella infection can lead to mild intestinal inflammation, which releases cytokines and other factors like interleukin-6 (IL-6), IL-8, IL-12, LPS-induced tumor necrosis factor alpha (LITAF) and interferon gamma (IFN-γ). The increases in expression of these pro-inflammatory cytokines affect bone metabolism, leading to bone loss (50). The above findings further corroborate the reliability of our analysis and prediction.

Further, the network analysis using the CytoHubba function in Cytoscape software identified ten hub genes. RT-qPCR results showed a significant increase in the mRNA expression levels of TNF, RARRES2, FLNA, MAP4K2, and SPI1 in the plasma of the OP patients group compared to the control group. Further, the mRNA expression levels of EGR2, JUNB, and CTSD were significantly decreased compared to the control sample group. Lastly, the mRNA expression levels of STXBP2 and NFKBIA were no significant difference. Previous studies have shown that TNF-α can act as an osteoclast factor, and TNF-β acts as an anti-osteoclast factor (4), which affects bone metabolism by regulating RANKL expression (3). Han L et al. (51) showed that RARRES2 protein secreted by adipocytes (52) has an inhibitory effect on osteoblast differentiation and proliferation by inhibiting Wnt/β-catenin signaling and activating RANK signaling. Osteocyte differentiation and proliferation are stimulatory. Therefore, maintaining low RARRES2 levels could be a strategic approach for OP prevention and treatment. Yang C et al. (53) showed that FLNA accumulates in the osteoblasts, and the osteoclasts were observed in the human OP samples. A report suggests that negative regulation of FLNA in mice is age-related and postmenopausal osteoporosis *in vitro* osteogenic differentiation in OP promotes RANKL-induced osteoclast differentiation (54). Zhang X et al. (55) showed an increase in MAP4K2 expression upstream of JNK in aged osteoblasts. Yang C et al. (56) showed the involvement ofSPI1 in OP development by regulating autophagy. Previous studies have shown that EGR2 is a zinc finger transcription factor, and EGFR signaling activates the MAPK/ERK pathway to stimulate EGR2 expression (57). Further, mounting evidence indicates that IL-27 inhibits RANKL-mediated osteoclast differentiation (8) in an EGR2-dependent manner (10). A previous report suggests that BMP-2-induced Smad1 protein activation leads to JUNB synthesis, which is involved in the trans-differentiation of myoblasts to osteoblasts and contributes to bone repair after OP (58). CTSD deficiency can lead to lysosomal autophagy, which plays a protective role in OP development (59). Previous studies show that STXBP2regulates vascular homeostasis in endothelial cells (60), along with various factors NFKBIA which significantly affect osteoclastogenesis (7, 61). However, in our study, there was no significant difference in the expression of STXBP2 and NFKBIA, as shown by RT-qPCR. Therefore, in the future, increasing the sample size would be a need for in-depth analysis. Taken together, it is suggested that the above molecules may play an important role in the diagnosis and treatment of OP.

In addition, for these eight OP-related differential genes, six diagnostic marker genes were tested by lasso regression and random forest using the new dataset. In recent years, osteoporosis treatment has focused on modulating the local immunity of the bone tissues. This provides a suitable microenvironment for positive regulation of bone metabolism, promotion of osteogenic differentiation, and inhibition of osteoclast differentiation (12). A report suggests that bone cells and the immune system share common progenitor cells, cytokines, and growth factors that interact during normal conditions and pathological states (3). However, the specific role of the immune system in OP is not fully understood. In this study, the CIBERSORT evaluated the immune cell infiltration in the two clusters. The results showed significant differences in monocytes, activated CD4 memory T cells, and memory and naïve B cells. Liu P et al (62) showed that monocytes express high levels of glucocorticoid receptors, which accumulate in the bone marrow during GC-induced osteoporosis, and have osteoclast differentiation potential. Gazzola L et al (63) revealed that higher levels of activated CD4+/CD8+ T cells are an independent predictor of osteopenia and osteoporosis. Titanji K et al (64) showed that individuals with HIV infection had significantly higher bone resorption and osteopenia, which were associated with B cell dysfunction. It is likely that a significant increase in RANKL-expressing B cells and a significant decrease in OPG-expressing B cells could be related to the induction of B cells naïve (46). Taken together, the immune microenvironment is under the tight regulation of cell-associated factors, which may play an important role in OP.

Moreover, two distinct molecular subtypes were identified using a consensus clustering approach based on six OP-related diagnostic marker genes. Prognostic analysis identified four key diagnostic marker genes (RARRES2, SLC4A2, MAP4K2, PPP1R15A). Among them, RARRES2, MAP4K2, and SPI1 could be used as hub genes, and significant difference in expression in OP have been established. ZYX can repair the vascular endothelial injury by regulating endothelial cell exocytosis to reorganize the local actin network (65). Previous studies have shown that SLC4A2-mediated osteoclast anion exchange affects bone resorption by regulating pHi (66, 67). PPP1R15A promotes apoptosis, alleviating stress-induced osteoblast damage (68, 69). Finally, interaction networks with miRNAs, TFs, RBPs, and drugs for key genes were constructed. The study’s results suggest that ten hub genes and six diagnostic marker genes could be used as diagnostic markers for OP.

However, this study has some obvious limitations. First, this study used bioinformatics analysis and proposed a theoretical diagnostic model. We have conducted a preliminary investigation to study the expression levels of Hub genes and pathway-related genes. A specific regulatory relationship needs to be further verified, for which a large sample size would be required to validate and enhance the clinical translational value of our diagnostic and prognostic model. Secondly, the immune characterization and cellular infiltration analysis were based on limited genetic data; thus, heterotypic cellular interactions and disturbances caused by different diseases may lead to bias in the immune analysis. Finally, further experimental validation using RT-qPCR, western blotting, and immunohistochemical analysis is required to fully understand the role of Hub genes and their underlying regulatory mechanisms associated with OP.

## Conclusion

In conclusion, we identified genes that may be differentially expressed in the OP and performed functional enrichment analysis on eight OP-related differential genes. CytoHubba function of Cytoscape software was used to conduct network analysis, and as a result, ten hub genes were identified. Further, RT-qPCR results confirmed that eight genes were significantly differential expressed, of which MAPK signaling pathway (p38, TRAF6), NF-kappa B signaling pathway (c-FLIP, MIP1β) were significantly differentially expressed between OP and control samples. The molecular features of OP prognosis based on six diagnostic marker genes were constructed. The immune infiltration analysis showed significant differences in monocytes, activated CD4 memory T cells, memory, and naïve B cells. Two different molecular subtypes of OP were identified using the consensus clustering method. Four key diagnostic marker genes were obtained from the prognostic analysis. Further, an interaction network with miRNA, TF, RBP, and drug was constructed for this purpose. We have identified a more accurate and reliable prognosis strategy for patients with early OP, which has enhanced our understanding of OP pathogenesis.

## Data availability statement

The datasets involved in the present study are available in the NCBI repository, accession numbers GSE56815, GSE7158, GSE56116 and GSE7429.

## Ethics statement

The studies involving human participants were reviewed and approved by the Second Affiliated Hospital of Inner Mongolia Medical University (ethical review number: YKD202002055). The patients/participants provided their written informed consent to participate in this study.

## Author contributions

XW, ZP, BZ and ZZ are responsible for the research concept and design. ZP and BZ wrote the manuscript. XW, ZP, TH, JA, SL, WH, XK, JC, ZZ, BZ participated in data collection and interpretation. All authors approved the final version of the manuscript. All authors contributed to the article and approved the submitted version.

## Funding

Science and Technology Planning Project of Inner Mongolia Science and Technology Department (2021GG0174; Inner Mongolia Education Department Project (NJZZ22665); Inner Mongolia Autonomous Region “14th Five-Year” social welfare field key research and development and achievement transformation plan project (2022YFSH0022); Science and Technology Planning Project of Inner Mongolia Science and Technology Department (2020GG0195); Inner Mongolia Autonomous Region “14th Five-Year” social welfare field key research and development and achievement transformation plan project (2022YFSH0021).

## Acknowledgments

Thanks to the Inner Mongolia Nature Foundation, Bayannaoer City Hospital, the Second Affiliated Hospital of Inner Mongolia Medical University.

## Conflict of interest

The authors declare that the research was conducted in the absence of any commercial or financial relationships that could be construed as a potential conflict of interest.

## Publisher’s note

All claims expressed in this article are solely those of the authors and do not necessarily represent those of their affiliated organizations, or those of the publisher, the editors and the reviewers. Any product that may be evaluated in this article, or claim that may be made by its manufacturer, is not guaranteed or endorsed by the publisher.

## References

[1] Zhang YL Chen Q Zheng L Zhang ZW Chen YJ Dai YC. Jianpi qingchang bushen decoction improves inflammatory response and metabolic bone disorder in inflammatory bowel disease-induced bone loss. World J Gastroenterol (2022) 28(13):1315–28. doi: 10.3748/wjg.v28.i13.1315 PMC9099185 35645540

[2] Lin S Wu J Chen B Li S Huang H. Identification of a potential MiRNA-mRNA regulatory network for osteoporosis by using bioinformatics methods: A retrospective study based on the gene expression omnibus database. Front Endocrinol (Lausanne) (2022) 13:844218. doi: 10.3389/fendo.2022.844218 35620387 PMC9128237

[3] Zhang W Zhao W Li W Geng Q Zhao R Yang Y. The imbalance of cytokines and lower levels of tregs in elderly Male primary osteoporosis. Front Endocrinol (Lausanne) (2022) 13:779264. doi: 10.3389/fendo.2022.779264 35721756 PMC9205399

[4] Sapra L Shokeen N Porwal K Saini C Bhardwaj A Mathew M. Bifidobacterium longum ameliorates ovariectomy-induced bone loss *via* enhancing anti-osteoclastogenic and immunomodulatory potential of regulatory b cells (Bregs). Front Immunol (2022) 13:875788. doi: 10.3389/fimmu.2022.875788 35693779 PMC9174515

[5] Zhang H Feng J Lin Z Wang S Wang Y Dai S. Identification and analysis of genes underlying bone mineral density by integrating microarray data of osteoporosis. Front Cell Dev Biol (2020) 8:798. doi: 10.3389/fcell.2020.00798 32974344 PMC7481435

[6] Marycz K Kowalczuk A Turlej E Zachanowicz E Tomaszewska A Kulpa-Greszta M. Impact of polyrhodanine manganese ferrite binary nanohybrids (PRHD@MnFe(2)O(4)) on osteoblasts and osteoclasts activities-a key factor in osteoporosis treatment. Mater (Basel) (2022) 15(11):3990. doi: 10.3390/ma15113990 PMC9181943 35683288

[7] Yu T Xiong Y Luu S You X Li B Xia J. The shared KEGG pathways between icariin-targeted genes and osteoporosis. Aging (Albany NY) (2020) 12(9):8191–201. doi: 10.18632/aging.103133 PMC7244047 32380477

[8] Changani H Parikh P. Molecular insights for an anti-osteoporotic properties of litsea glutinosa on saos-2 cells: An *in-vitro* approach. J Ayurveda Integr Med (2021) 13(2):100501. doi: 10.1016/j.jaim.2021.07.017 34799209 PMC8728066

[9] He Q Yang J Chen D Li Y Gong D Ge H. 12-Deoxyphorbol-13-Hexadecanoate abrogates OVX-induced bone loss in mice and osteoclastogenesis *via* inhibiting ROS level and regulating RANKL-mediated NFATc1 activation. Front Pharmacol (2022) 13:899776. doi: 10.3389/fphar.2022.899776 35721216 PMC9204068

[10] Shukla P Mansoori MN Kakaji M Shukla M Gupta SK Singh D. Interleukin 27 (IL-27) alleviates bone loss in estrogen-deficient conditions by induction of early growth response-2 gene. J Biol Chem (2017) 292(11):4686–99. doi: 10.1074/jbc.M116.764779 PMC5377783 28130449

[11] Wang X Liu X He P Guan K Yang Y Lei Y. The imbalance of mitochondrial homeostasis of peripheral blood-derived macrophages mediated by MAFLD may impair the walking ability of elderly patients with osteopenia. Oxid Med Cell Longev (2022) 2022:5210870. doi: 10.1155/2022/5210870 35368864 PMC8970807

[12] Zheng L Zhuang Z Li Y Shi T Fu K Yan W. Bone targeting antioxidative nano-iron oxide for treating postmenopausal osteoporosis. Bioact Mater (2022) 14:250–61. doi: 10.1016/j.bioactmat.2021.11.012 PMC8897644 35310348

[13] Zhou Y Gao Y Xu C Shen H Tian Q Deng HW. A novel approach for correction of crosstalk effects in pathway analysis and its application in osteoporosis research. Sci Rep (2018) 8(1):668. doi: 10.1038/s41598-018-19196-2 29330445 PMC5766601

[14] Chen J Wang L Shen Y Yu J Ye T Zhuang C. Key genes associated with osteoporosis revealed by genome wide gene expression analysis. Mol Biol Rep (2014) 41(9):5971–7. doi: 10.1007/s11033-014-3474-1 24993113

[15] Xu F Gao F. Liuwei dihuang pill cures postmenopausal osteoporosis with kidney-yin deficiency: Potential therapeutic targets identified based on gene expression profiling. Med (Baltimore) (2018) 97(31):e11659. doi: 10.1097/MD.0000000000011659 PMC6081159 30075554

[16] Kazezian Z Gawri R Haglund L Ouellet J Mwale F Tarrant F. Gene expression profiling identifies interferon signalling molecules and IGFBP3 in human degenerative annulus fibrosus. Sci Rep (2015) 5:15662. doi: 10.1038/srep15662 26489762 PMC4614807

[17] Safran M Dalah I Alexander J Rosen N Iny Stein T Shmoish M. GeneCards version 3: the human gene integrator. (2010) 2010:baq020 doi: 10.1093/database/baq020 PMC2938269 20689021

[18] Wu S Liu S Chen N Zhang C Zhang H Guo X. Genome-wide identification of immune-related alternative splicing and splicing regulators involved in abdominal aortic aneurysm. Front Genet (2022) 13:816035. doi: 10.3389/fgene.2022.816035 35251127 PMC8892299

[19] Whitehead MJ McCanney GA Willison HJ Barnett SC. MyelinJ: an ImageJ macro for high throughput analysis of myelinating cultures. Bioinformatics 35(21):4528–30. doi: 10.1093/bioinformatics/btz403. PMC6821319 31095292

[20] Newman AM Steen CB Liu CL Gentles AJ Chaudhuri AA Scherer F. Determining cell type abundance and expression from bulk tissues with digital cytometry. Nat Biotechnol (2019) 37(7):773–82. doi: 10.1038/s41587-019-0114-2 PMC6610714 31061481

[21] Wu X Sui Z Zhang H Wang Y Yu Z. Integrated analysis of lncrna-mediated ceRNA network in lung adenocarcinoma. Front Oncol (2020) 10:554759. doi: 10.3389/fonc.2020.554759 33042838 PMC7523091

[22] Wang J Cong S Wu H He Y Liu X Sun L. Identification and analysis of potential autophagy-related biomarkers in endometriosis by WGCNA. Front Mol Biosci (2005) 8:743012. doi: 10.3389/fmolb.2021.743012 PMC8591037 34790699

[23] Tian X Liu B Chen L Xie Y Liang J Yang Y. RNA-seq identifies marked th17 cell activation and altered cftr expression in different atopic dermatitis subtypes in chinese han populations. Front Immunol (2015) 12:628512. doi: 10.3389/fimmu.2021.628512 PMC8047326 33868246

[24] Langfelder P Horvath S. WGCNA: an r package for weighted correlation network analysis. BMC Bioinformatics (2008) 9(1):1–13. doi: 10.1186/1471-2105-9-559 19114008 PMC2631488

[25] Harris MA Clark J Ireland A Lomax J Ashburner M Foulger R Gene Ontology Consortium. The gene ontology (GO) database and informatics resource. Nucleic Acids Res (2004) 32(suppl_1):D258–D61. doi: 10.1093/nar/gkh036 PMC308770 14681407

[26] Kanehisa M Goto SJNar. KEGG. Kyoto encyclopedia of genes and genomes. Nucleic Acids Res (2000) 28(1):27–30. doi: 10.1093/nar/28.1.27 10592173 PMC102409

[27] Wu T Hu E Xu S Chen M Guo P Dai Z. clusterProfiler 4. : A Universal Enrichment Tool Interpreting Omics Data (2021) 2(3):100141. doi: 10.1016/j.xinn.2021.100141 PMC8454663 34557778

[28] Yu G Wang LG Yan GR He QY. DOSE: an R/Bioconductor package for disease ontology semantic and enrichment analysis. Bioinformatics (2015) 31(4):608–9. doi: 10.1093/bioinformatics/btu684 25677125

[29] Subramanian A Tamayo P Mootha VK Mukherjee S Ebert BL Gillette MA. Gene set enrichment analysis: a knowledge-based approach for interpreting genome-wide expression profiles. Proc Natl Acad Sci U S A (2005) 102(43):15545–50. doi: 10.1073/pnas.0506580102 PMC1239896 16199517

[30] Liberzon A Birger C Thorvaldsdottir H Ghandi M Mesirov JP Tamayo P. The molecular signatures database (MSigDB) hallmark gene set collection. Cell Syst (2015) 1(6):417–25. doi: 10.1016/j.cels.2015.12.004 PMC4707969 26771021

[31] Hänzelmann S Castelo R Guinney JJBb. GSVA. Gene set variation analysis for microarray and RNA-seq data. BMC Bioinformatics (2013) 14(1):1–15. doi: 10.1186/1471-2105-14-7 23323831 PMC3618321

[32] Yang C Ren J Li B Jin C Ma C Cheng C. Identification of gene biomarkers in patients with postmenopausal osteoporosis. Mol Med Rep (2019) 19(2):1065–73. doi: 10.3892/mmr.2018.9752 PMC6323213 30569177

[33] Robin X Turck N Hainard A Tiberti N Lisacek F Sanchez J-C. pROC: an open-source package for r and s+ to analyze and compare ROC curves. BMC Bioinformatics (2011) 12(1):1–8. doi: 10.1186/1471-2105-12-77 21414208 PMC3068975

[34] Von Mering C Jensen LJ Snel B Hooper SD Krupp M Foglierini M. STRING: known and predicted protein–protein associations, integrated and transferred across organisms. Nucleic Acids Res. (2005) 33(suppl_1):D433–D7. doi: 10.1093/nar/gki005 PMC539959 15608232

[35] Shannon P Markiel A Ozier O Baliga NS Wang JT Ramage D. Cytoscape: a software environment for integrated models of biomolecular interaction networks. Genome Res (2003) 13(11):2498–504. doi: 10.1101/gr.1239303 PMC403769 14597658

[36] Chin C-H Chen S-H Wu H-H Ho C-W Ko M-T Lin C-Y. cytoHubba: identifying hub objects and sub-networks from complex interactome. BMC Syst Biol (2014) 8(4):1–7. doi: 10.1186/1752-0509-8-S4-S11 25521941 PMC4290687

[37] Engebretsen S Bohlin J. Statistical predictions with glmnet. Clin Epigenetics (2019) 11(1):123. doi: 10.1186/s13148-019-0730-1 31443682 PMC6708235

[38] Fang Y Huang S Han L Wang S Xiong B. Package ‘forestplot’. Cancer Manag Res (2021) 13:5599–611. doi: 10.2147/CMAR.S318704 PMC8285530 34285580

[39] Liu TT Li R Huo C Li JP Yao J Ji XL. Comprehensive analysis of peritoneal metastasis sequencing data to identify linc00924 as a prognostic biomarker in gastric cancer. Front Cell Dev Biol (2021) 9:682002. doi: 10.3389/fcell.2021.682002 34409029 PMC8366777

[40] Zhang H Meltzer P Davis S. RCircos: an r package for circos 2D track plots. BMC Bioinf (2013) 14:244. doi: 10.1186/1471-2105-14-244 PMC3765848 23937229

[41] Cunningham F Achuthan P Akanni W Allen J Amode MR Armean IM. Ensembl 2019. Nucleic Acids Res (2019) 47(D1):D745–d51. doi: 10.1093/nar/gky1113 PMC6323964 30407521

[42] Tang Z Kang B Li C Chen T Zhang Z. GEPIA2: an enhanced web server for large-scale expression profiling and interactive analysis. Nucleic Acids Res (2019) 47(W1):W556–W60. doi: 10.1093/nar/gkz430 PMC6602440 31114875

[43] Chang L Zhou G Soufan O Xia JJNAR. miRNet 2.0: network-based visual analytics for miRNA functional analysis and systems biology. Nucleic Acids Res (2020) 48(W1):W244–W51. doi: 10.1093/nar/gkaa467 PMC7319552 32484539

[44] Caudron-Herger M Jansen RE Wassmer E Diederichs S. RBP2GO: a comprehensive pan-species database on RNA-binding proteins, their interactions and functions. Nucleic Acids Res (2021) 49(D1):D425–D36. doi: 10.1093/nar/gkaa1040 PMC7778890 33196814

[45] Dong Q Li F Xu Y Xiao J Xu Y Shang D. RNAactDrug: a comprehensive database of RNAs associated with drug sensitivity from multi-omics data. Brief Bioinform (2020) 21(6):2167–74. doi: 10.1093/bib/bbz142 31799597

[46] Titanji K Ofotokun I Weitzmann MN. Immature/transitional b-cell expansion is associated with bone loss in HIV-infected individuals with severe CD4+ T-cell lymphopenia. Aids (2020) 34(10):1475–83. doi: 10.1097/QAD.0000000000002563 PMC7371241 32675561

[47] Tao H Tao Y Yang C Li W Zhang W Li X. Gut metabolite urolithin a inhibits osteoclastogenesis and senile osteoporosis by enhancing the autophagy capacity of bone marrow macrophages. Front Pharmacol (2022) 13:875611. doi: 10.3389/fphar.2022.875611 35645801 PMC9135380

[48] Liu T Jiang L Xiang Z Li J Zhang Y Xiang T. Tereticornate a suppresses RANKL-induced osteoclastogenesis *via* the downregulation of c-src and TRAF6 and the inhibition of RANK signaling pathways. BioMed Pharmacother (2022) 151:113140. doi: 10.1016/j.biopha.2022.113140 35605290

[49] Kim H Lee K Kim JM Kim MY Kim JR Lee HW. Selenoprotein W ensures physiological bone remodeling by preventing hyperactivity of osteoclasts. Nat Commun (2021) 12(1):2258. doi: 10.1038/s41467-021-22565-7 33859201 PMC8050258

[50] Raehtz S Hargis BM Kuttappan VA Pamukcu R Bielke LR McCabe LR. High molecular weight polymer promotes bone health and prevents bone loss under salmonella challenge in broiler chickens. Front Physiol (2018) 9:384. doi: 10.3389/fphys.2018.00384 29706903 PMC5908899

[51] Han L Zhang Y Wan S Wei Q Shang W Huang G. Loss of chemerin triggers bone remodeling *in vivo* and *in vitro* . Mol Metab (2021) 53:101322. doi: 10.1016/j.molmet.2021.101322 34416393 PMC8450264

[52] Guo Y Huo J Wu D Hao H Ji X Zhao E. Simvastatin inhibits the adipogenesis of bone marrow−derived mesenchymal stem cells through the downregulation of chemerin/CMKLR1 signaling. Int J Mol Med (2020) 46(2):751–61. doi: 10.3892/ijmm.2020.4606 PMC7307816 32468037

[53] Yang C Yang P Liu P Wang H Ke E Li K. Targeting filamin a alleviates ovariectomy-induced bone loss in mice *via* the WNT/β-catenin signaling pathway. Cell Signal (2022) 90:110191. doi: 10.1016/j.cellsig.2021.110191 34774991

[54] Goldberg S Glogauer J Grynpas MD Glogauer M. Deletion of filamin a in monocytes protects cortical and trabecular bone from post-menopausal changes in bone microarchitecture. Calcif Tissue Int (2015) 97(2):113–24. doi: 10.1007/s00223-015-9994-4 25894069

[55] Zhang X Zhao G Zhang Y Wang J Wang Y Cheng L. Activation of JNK signaling in osteoblasts is inversely correlated with collagen synthesis in age-related osteoporosis. Biochem Biophys Res Commun (2018) 504(4):771–6. doi: 10.1016/j.bbrc.2018.08.094 30217450

[56] Yang C Tao H Zhang H Xia Y Bai J Ge G. TET2 regulates osteoclastogenesis by modulating autophagy in OVX-induced bone loss. Autophagy (2022) 24:1–13. doi: 10.1080/15548627.2022.2048432 PMC9673923 35255774

[57] Chandra A Lan S Zhu J Siclari VA Qin L. Epidermal growth factor receptor (EGFR) signaling promotes proliferation and survival in osteoprogenitors by increasing early growth response 2 (EGR2) expression. J Biol Chem (2013) 288(28):20488–98. doi: 10.1074/jbc.M112.447250 PMC3711314 23720781

[58] Lin DPL Carnagarin R Dharmarajan A Dass CR. Transdifferentiation of myoblasts into osteoblasts - possible use for bone therapy. J Pharm Pharmacol (2017) 69(12):1661–71. doi: 10.1111/jphp.12790 28809431

[59] Deng YX He WG Cai HJ Jiang JH Yang YY Dan YR. Analysis and validation of hub genes in blood monocytes of postmenopausal osteoporosis patients. Front Endocrinol (Lausanne) (2021) 12:815245. doi: 10.3389/fendo.2021.815245 35095774 PMC8792966

[60] Schillemans M Karampini E Hoogendijk AJ Wahedi M van Alphen FPJ van den Biggelaar M. Interaction networks of weibel-palade body regulators syntaxin-3 and syntaxin binding protein 5 in endothelial cells. J Proteom (2019) 205:103417. doi: 10.1016/j.jprot.2019.103417 31201948

[61] Yang L Zhang B Liu J Dong Y Li Y Li N. Protective effect of acteoside on ovariectomy-induced bone loss in mice. Int J Mol Sci (2019) 20(12):2974. doi: 10.3390/ijms20122974 PMC6627387 31216684

[62] Liu P Gao Y Luo P Yu H Guo S Liu F. Glucocorticoid-induced expansion of classical monocytes contributes to bone loss. Exp Mol Med (2022) 54(6):765–76. doi: 10.1038/s12276-022-00764-6 PMC9256622 35672449

[63] Gazzola L Bellistri GM Tincati C Ierardi V Savoldi A Del Sole A. Association between peripheral T-lymphocyte activation and impaired bone mineral density in HIV-infected patients. J Transl Med (2013) 11:51. doi: 10.1186/1479-5876-11-51 23448662 PMC3598927

[64] Titanji K Vunnava A Sheth AN Delille C Lennox JL Sanford SE. Dysregulated b cell expression of RANKL and OPG correlates with loss of bone mineral density in HIV infection. PloS Pathog (2014) 10(10):e1004497. doi: 10.1371/journal.ppat.1004497 25393853 PMC4231117

[65] Han X Li P Yang Z Huang X Wei G Sun Y. Zyxin regulates endothelial von willebrand factor secretion by reorganizing actin filaments around exocytic granules. Nat Commun (2017) 8:14639. doi: 10.1038/ncomms14639 28256511 PMC5338022

[66] Xue JY Grigelioniene G Wang Z Nishimura G Iida A Matsumoto N. SLC4A2 deficiency causes a new type of osteopetrosis. J Bone Miner Res (2022) 37(2):226–35. doi: 10.1002/jbmr.4462 34668226

[67] Wu C Liu X Sun R Qin Y Liu Z Yang S. Targeting anion exchange of osteoclast, a new strategy for preventing Wear particles induced- osteolysis. Front Pharmacol (2018) 9:1291. doi: 10.3389/fphar.2018.01291 30459624 PMC6232501

[68] Chang IC Chiang TI Lo C Lai YH Yue CH Liu JY. Anemone altaica induces apoptosis in human osteosarcoma cells. Am J Chin Med (2015) 43(5):1031–42. doi: 10.1142/S0192415X15500597 26224029

[69] Lin CC Chao PY Shen CY Shu JJ Yen SK Huang CY. Novel target genes responsive to apoptotic activity by ocimum gratissimum in human osteosarcoma cells. Am J Chin Med (2014) 42(3):743–67. doi: 10.1142/S0192415X14500487 24871663

