# Supplementary material for: Prognostic analysis and validation of diagnostic marker genes in patients with osteoporosis
Source: Front Immunol. 2022 Oct 13;13:987937. doi: 10.3389/fimmu.2022.987937 (PMC9610549; doi:10.3389/fimmu.2022.987937)
Supplement: Supplementary file 1 [file DataSheet_1.docx]

**Figures legends**

**Figure S1. Weighted gene co-expression network analysis (WGCNA) analysis.** S1A: WGCNA threshold screening. S1B: Clustering of co-expressed genes. S1C: Correlation of gene clusters with sample disease. S1D: Association analysis of the most significant gene clusters with osteoporosis.

**Figure S2. Protein-Protein Interaction (PPI) network.** A: PPI network of key genes B: PPI network hub gene subnet, the darker the red color, the higher the degree of the gene. C: Association of the validated diagnostic genes.

**Figure S3. Association network of key genes and miRNAs.** The publicly available databases were used to identify miRNAs, TF, RBPs, and drugs associated with each key gene to explore key gene-related association factors and draw respective association networks. The miRNet database provided the regulatory relationship between mRNA and microRNA (miRNA). Construction of OP key gene-miRNA regulatory network based on miRNA-gene regulatory relationship (Figure S2.A-D).

**Figure S4. Association network of key genes and TFs.** The miRNet database provides the regulatory relationship between mRNA, microRNA (miRNA), and TF. the TFs associated with two key genes (SLC4A2 and SPI1) were obtained. The OP key gene-transcription factor regulatory network was constructed based on the TF-gene regulatory relationship (Figure S3).

**Figure S5. Association network of key genes and RBPs.** The gene-RBP regulatory relationship was obtained from the RBP2GO database and was used to construct the regulatory network of OP key genes-RBP (Figure S4.A-D).

**Figure S6. Association network of key genes and drugs.** The drug-gene regulatory relationship obtained from the RNAactDrug database was used to construct a regulatory network of key OP genes-drugs (Figure S5.A-D).

Table S1. Differential gene expression list

| OP-related differential genes（17） | OP-related differential key genes（8） |
| --- | --- |
| MAP4K2、FLNA、JUNB、PPP1R15A、STXBP2、CTSD、ZYX、SPI1、SLC4A2、RARRES2、NFKBIA、APOE、TNF、CLIC2、MAT1A、TTR、EGR2 | EGR2, RARRES2, ZYX, SLC4A2, MAP4K2, MAT1A, PPP1R15A, SPI1 |

Table S2. Data set information table

|  | GSE56815 | GSE7158 | GSE56116 | GSE7429 |
| --- | --- | --- | --- | --- |
| **Organism** | Homo sapiens | Homo sapiens | Homo sapiens | Homo sapiens |
| **Experiment type** | Expression profiling by array | Expression profiling by array | Expression profiling by array | Expression profiling by array |
| **Platforms** | GPL96 | GPL570 | GPL1433 | GPL96 |
| **Sample(number)** | 80 | 26 | 13 | 20 |
| **Sample of normal(number)** | 40 | 14 | 3 | 10 |
| **Sample of OP(number)** | 40 | 12 | 10 | 10 |

Table S3. GO enrichment analysis results

| Description | ONTOLOGY | p.adjust | qvalue |
| --- | --- | --- | --- |
| activation of MAPKKK activity | BP | 0.015023579 | 0.008259515 |
| maintenance of location | BP | 0.015023579 | 0.008259515 |
| positive regulation of stress-activated MAPK cascade | BP | 0.015023579 | 0.008259515 |
| positive regulation of stress-activated protein kinase signaling cascade | BP | 0.015023579 | 0.008259515 |
| digestive tract development | BP | 0.015023579 | 0.008259515 |
| platelet degranulation | BP | 0.015023579 | 0.008259515 |
| negative regulation of lipid storage | BP | 0.015023579 | 0.008259515 |
| cytoplasmic sequestering of protein | BP | 0.015023579 | 0.008259515 |
| digestive system development | BP | 0.015023579 | 0.008259515 |
| myeloid cell differentiation | BP | 0.021970555 | 0.012078755 |
| positive regulation of translational initiation | BP | 0.021970555 | 0.012078755 |
| embryonic digestive tract development | BP | 0.021970555 | 0.012078755 |
| regulation of stress-activated MAPK cascade | BP | 0.024613735 | 0.013531897 |
| regulation of stress-activated protein kinase signaling cascade | BP | 0.024613735 | 0.013531897 |
| positive regulation of pri-miRNA transcription by RNA polymerase II | BP | 0.029141581 | 0.016021171 |
| myeloid leukocyte differentiation | BP | 0.029141581 | 0.016021171 |
| fat cell differentiation | BP | 0.034651868 | 0.019050562 |
| regulation of pri-miRNA transcription by RNA polymerase II | BP | 0.039218582 | 0.021561206 |
| pri-miRNA transcription by RNA polymerase II | BP | 0.039218582 | 0.021561206 |
| regulation of lipid storage | BP | 0.039258849 | 0.021583343 |

Table S4. KEGG enrichment analysis results

| Description | p.adjust | qvalue |
| --- | --- | --- |
| Osteoclast differentiation | 0.001710452 | 0.001076573 |
| Human T-cell leukemia virus 1 infection | 0.007395261 | 0.00465464 |
| C-type lectin receptor signaling pathway | 0.00934603 | 0.005882471 |
| TNF signaling pathway | 0.00934603 | 0.005882471 |
| Apoptosis | 0.013213942 | 0.008316968 |
| Hepatitis B | 0.018321122 | 0.011531472 |
| Legionellosis | 0.035217963 | 0.022166488 |

Table S5. GSEA enrichment analysis results (GO enrichment results)

| Description | enrichmentScore | p.adjust | qvalues |
| --- | --- | --- | --- |
| negative regulation of cellular process | 0.596145811 | 0.001538462 | 0.090620251 |
| negative regulation of metabolic process | 0.644527796 | 0.001574803 | 0.090620251 |
| regulation of response to stimulus | 0.556014053 | 0.004739336 | 0.119138426 |
| negative regulation of macromolecule metabolic process | 0.604400782 | 0.004830918 | 0.119138426 |
| negative regulation of cellular metabolic process | 0.60436578 | 0.004830918 | 0.119138426 |
| negative regulation of nitrogen compound metabolic process | 0.60436578 | 0.004830918 | 0.119138426 |
| response to stress | 0.49885618 | 0.009230769 | 0.174218907 |
| positive regulation of biosynthetic process | 0.590423987 | 0.01255887 | 0.174218907 |
| positive regulation of macromolecule biosynthetic process | 0.590423987 | 0.01255887 | 0.174218907 |
| positive regulation of cellular biosynthetic process | 0.590423987 | 0.01255887 | 0.174218907 |
| response to chemical | 0.468290235 | 0.013636364 | 0.174218907 |
| phosphorus metabolic process | 0.578177471 | 0.014128728 | 0.174218907 |
| phosphate-containing compound metabolic process | 0.578177471 | 0.014128728 | 0.174218907 |
| regulation of immune system process | 0.567486316 | 0.018838305 | 0.216805751 |
| response to organic substance | 0.464057145 | 0.020537125 | 0.221584768 |
| regulation of catalytic activity | 0.544706284 | 0.022047244 | 0.223885327 |
| cellular response to chemical stimulus | 0.455088664 | 0.04552352 | 0.319254943 |
| regulation of developmental process | 0.478245599 | 0.047913447 | 0.319254943 |
| negative regulation of biological process | 0.596145811 | 0.001538462 | 0.090620251 |

Table S6-1. GSVA enrichment analysis results

| Description | logFC | P.Value | adj.P.Val |
| --- | --- | --- | --- |
| go_cell_killing | 0.650829563 | 0.018529 | 0.207261546 |
| go_heart_process | -0.819769308 | 0.001292999 | 0.033084609 |
| go_snare_binding | 0.650829563 | 0.018529 | 0.207261546 |
| go_whole_membrane | 0.763226493 | 8.02E-05 | 0.004102014 |
| go_detoxification | -0.846603158 | 0.000270899 | 0.011798501 |
| go_gtpase_binding | 0.820072229 | 0.001527582 | 0.037674211 |
| go_golgi_membrane | 0.731684982 | 0.011078137 | 0.18587661 |
| go_gtpase_activity | 0.964770524 | 0.000394581 | 0.013241091 |
| go_protein_folding | 0.660353693 | 0.007788936 | 0.151847164 |
| go_kinase_activity | 0.731684982 | 0.011078137 | 0.18587661 |
| go_vesicle_docking | 0.650829563 | 0.018529 | 0.207261546 |
| go_zymogen_granule | 0.650829563 | 0.018529 | 0.207261546 |
| go_tube_development | 0.679228285 | 0.000555816 | 0.017503928 |
| go_cation_transport | -0.819769308 | 0.001292999 | 0.033084609 |
| go_membrane_docking | 0.650829563 | 0.018529 | 0.207261546 |
| go_exocytic_process | 0.650829563 | 0.018529 | 0.207261546 |
| go_syntaxin_binding | 0.650829563 | 0.018529 | 0.207261546 |
| go_apoptotic_process | 0.747009062 | 2.74E-05 | 0.001439434 |
| go_vacuolar_membrane | 0.800706591 | 0.000772531 | 0.023602563 |
| go_vesicle_targeting | 0.731684982 | 0.011078137 | 0.18587661 |

Table S6-2. GSVA enrichment analysis results (KEGG results)

| Description | logFC | P.Value | adj.P.Val |
| --- | --- | --- | --- |
| kegg_ubiquitin_mediated_proteolysis | 0.667797888 | 0.020629498 | 0.656648205 |

Table S7-1. Primers for RT-qPCR (hub gene)

| **gene name** | **Primer sequence（5’-3’）** | |
| --- | --- | --- |
| Human TNF | F | GGCAGGTCTACTTTGGAGTCATTGC |
|  | R | ACATTCGAGGCTCCAGTGAATTCGG |
| Human RARRES2 | F | TGGAAGAAACCCGAGTGCAAA |
|  | R | AGAACTTGGGTCTCTATGGGG |
| Human FLNA | F | CTTATCGCGCTGTTGGAGGT |
|  | R | GCCACCGACACGTTCTCAA |
| Human STXBP2 | F | ATTCTGAGCGGAGTTATTCGGA |
|  | R | CCGCCGTTTGTTGATGTCTTC |
| Human EGR2 | F | CCTGCGACCTCGAAAGTA |
|  | R | TCGTCACTCCTGGCAAAC |
| Human MAP4K2 | F | GGAGCGGCAGATTGCCTAC |
|  | R | GTCCCAATGAAAGACCTCCTCT |
| Human NFKBIA | F | ACCTGGTGTCACTCCTGTTGA |
|  | R | CTGCTGCTGTATCCGGGTG |
| Human JUNB | F | ACTCATACACAGCTACGGGATACG |
|  | R | GGCTCGGTTTCAGGAGTTTG |
| Human Spi1 | F | ATGTTACAGGCGTGCAAAATGG |
|  | R | TGATCGCTATGGCTTTCTCCA |
| Human CTSD | F | CAGCCAGGCATCACCTTCAT |
|  | R | CAGGTAGAAGGAGAAGATGT |
| Human GAPDH | F | GAAGGTGAAGGTCGGAGTC |
|  | R | GAAGATGGTGATGGGATTTC |

Table S7-2. Primers used for RT-qPCR (pathway genes)

| **gene name** | **Primer sequence（5’-3’）** | |
| --- | --- | --- |
| Human c-FLIP | F | CGGACTATAGAGTGCTGATGG |
|  | R | GATTATCAGGCAGATTCCTAG |
| Human MIP1β | F | CTGTGCTGATCCCAGTGAATC |
|  | R | TCAGTTCAGTTCCAGGTCATACA |
| Human p38 | F | TCAGTCCATCATTCATGCGAAA |
|  | R | AACGTCCAACAGACCAATCAC |
| Human TRAF6 | F | TTTGCTCTTATGGATTGTCCCC |
|  | R | CATTGATGCAGCACAGTTGTC |
| Human GAPDH | F | GAAGGTGAAGGTCGGAGTC |
|  | R | GAAGATGGTGATGGGATTTC |
