# Supplementary figures and images for: Prognostic analysis and validation of diagnostic marker genes in patients with osteoporosis
Source: Front Immunol. 2022 Oct 13;13:987937. doi: 10.3389/fimmu.2022.987937 (PMC9610549; doi:10.3389/fimmu.2022.987937)

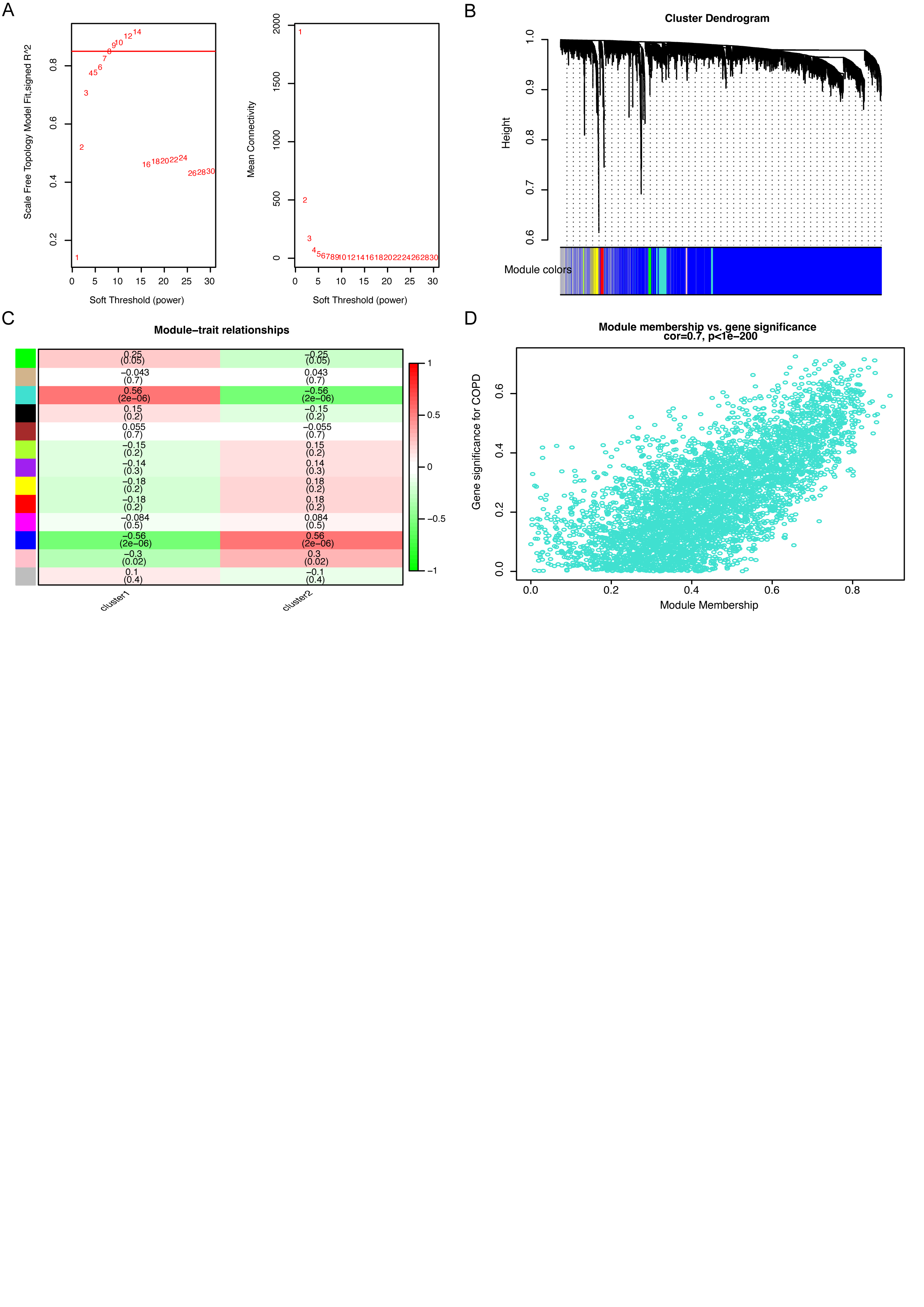

Supplement: Supplementary file 2 [file Image_1.tif]

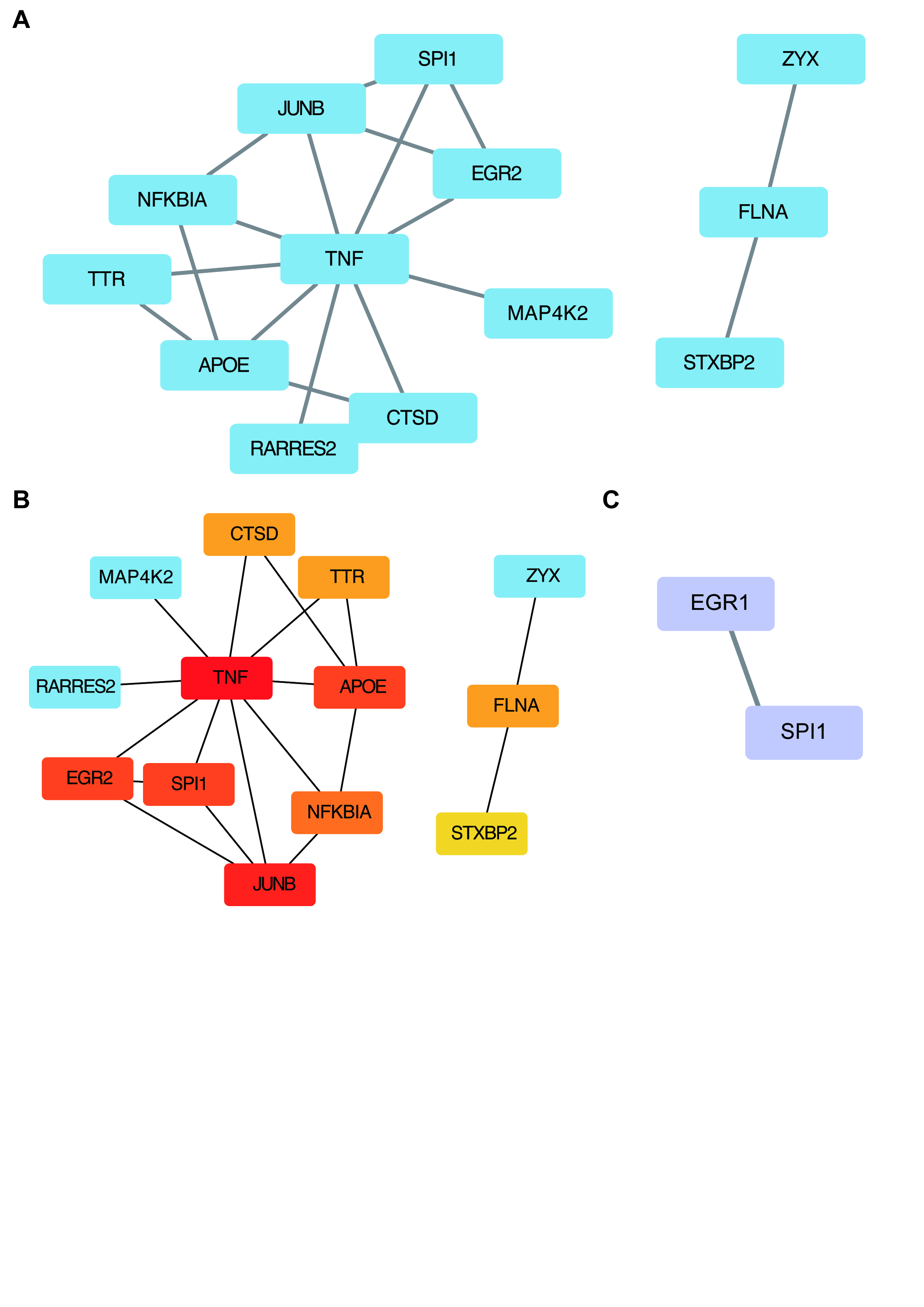

Supplement: Supplementary file 3 [file Image_2.tif]

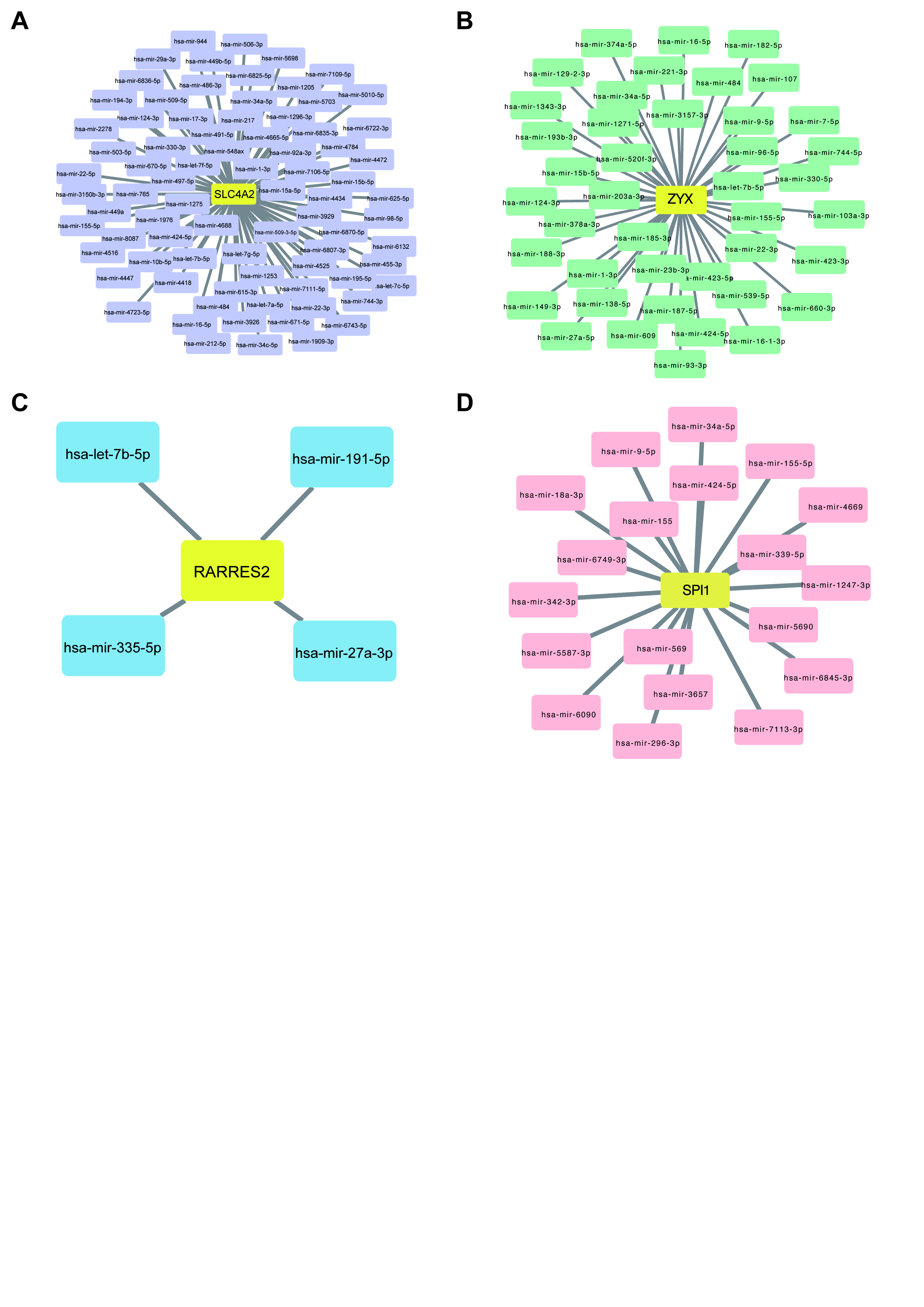

Supplement: Supplementary file 4 [file Image_3.tif]

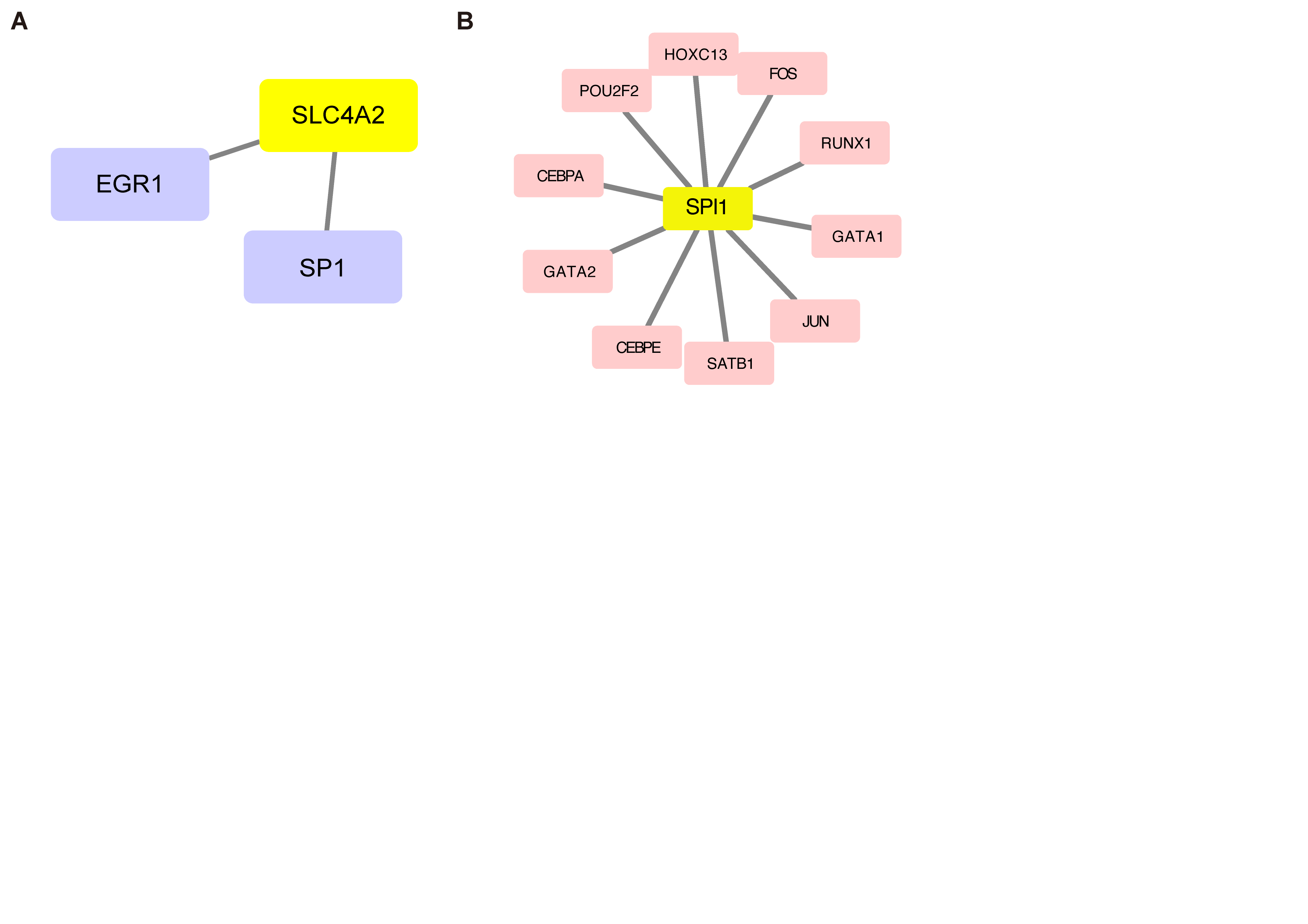

Supplement: Supplementary file 5 [file Image_4.tif]

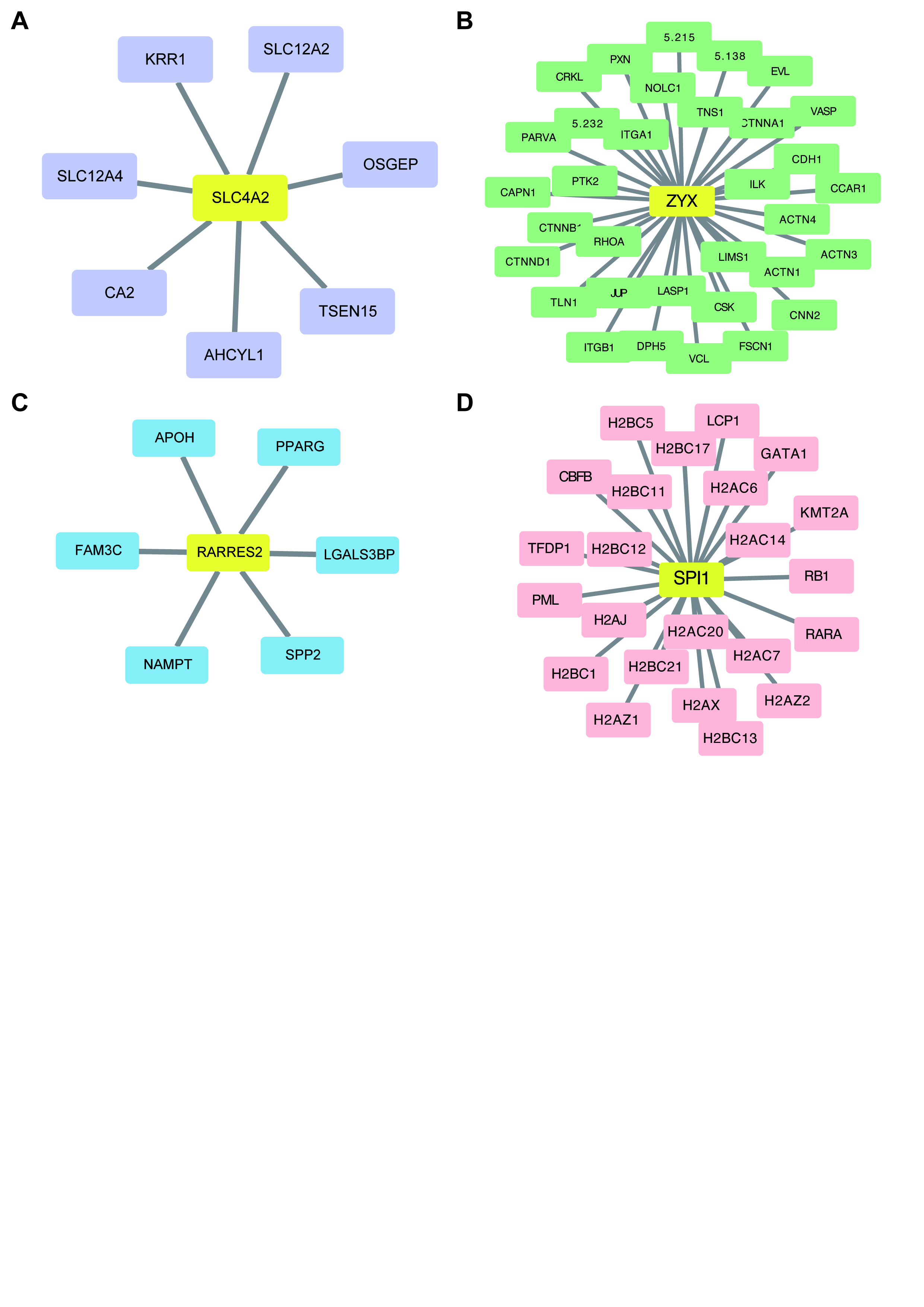

Supplement: Supplementary file 6 [file Image_5.tif]

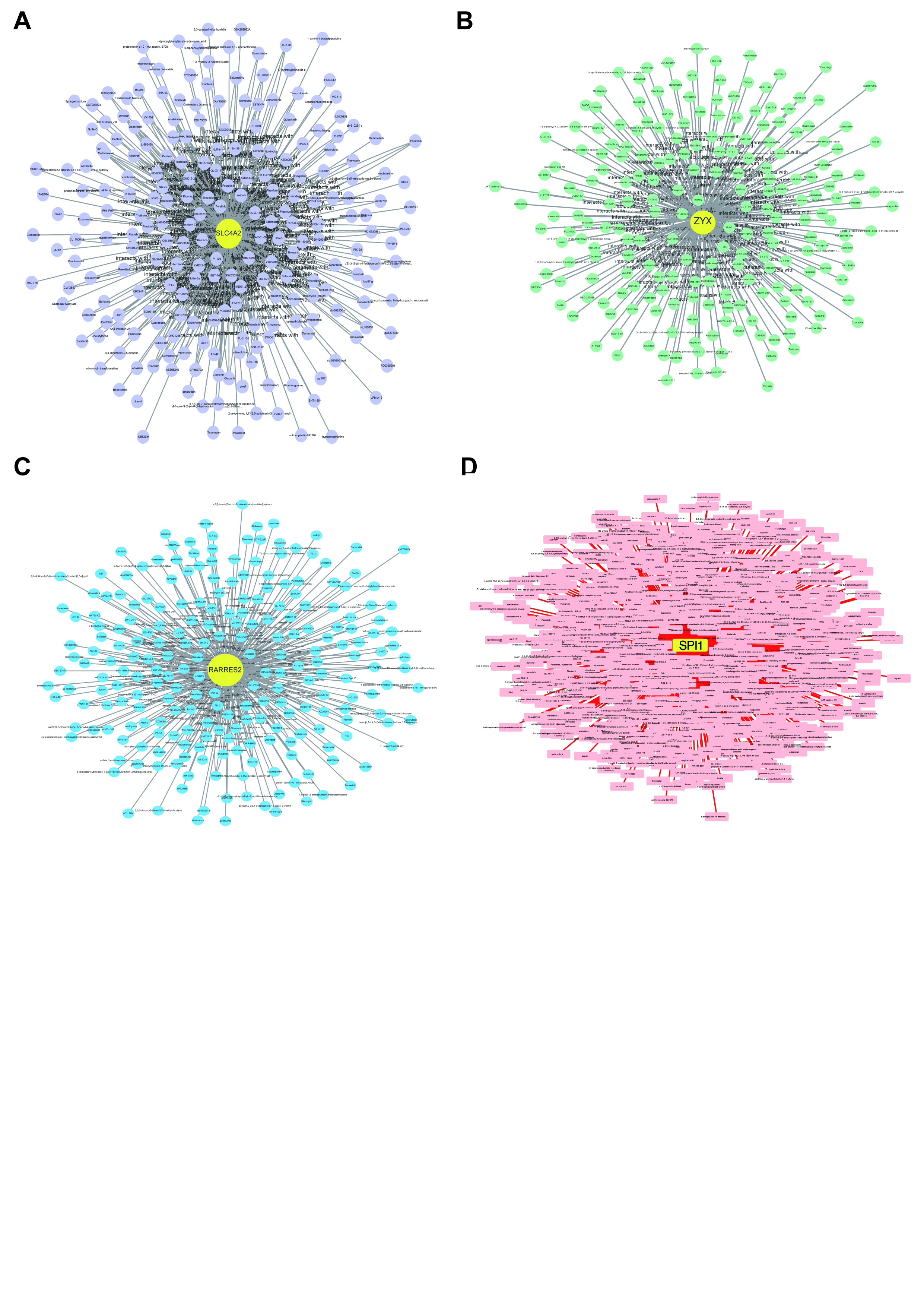

Supplement: Supplementary file 7 [file Image_6.tif]
